# Anti-Inflammatory Effects of Fermented Bark of *Acanthopanax sessiliflorus* and Its Isolated Compounds on Lipopolysaccharide-Treated RAW 264.7 Macrophage Cells

**DOI:** 10.1155/2020/6749425

**Published:** 2020-07-21

**Authors:** Min Ji Kim, Hye Soo Wang, Min Won Lee

**Affiliations:** Laboratory of Pharmacognosy and Natural Product Derived Medicine, College of Pharmacy, Chung-Ang University, Seoul 06974, Republic of Korea

## Abstract

The fermentation was carried out on the bark of *Acanthopanax sessiliflorus* (AS). *Acanthopanax* species have been used in traditional medicine as tonics, sedatives, and antispasmodics. An activity-guided isolation of the fermented bark of *A. sessiliflorus* (FAS) yielded several phytochemicals: acanthoside D (**1**), acanthoside B (**2**), daucosterol (**3**), protocatechuic acid (**4**), chlorogenic acid methyl ester (**5**), ciwujiatone (**6**), syringaresinol (**7**), farnesol (**8**), 3,4-dicaffeoylquinic acid (**9**), and falcarindiol (**10**). HPLC analysis showed that content of lignan glycoside (**1)** was decreased and **4** and **7** were increased after fermentation. Anti-inflammatory activities on FAS showed the decrease of nitric oxide (NO) production, and inhibitory activities of iNOS and COX-2, proinflammatory cytokines (IL-6 and tumor necrosis factor-*α*), and collagenase. The aglycone, syringaresinol (**7**), which was increased through fermentation showed enhanced activity than **1**. Thus, FAS may have the potential to treat inflammatory disorders, such as arthritis.

## 1. Introduction

*Acanthopanax sessiliflorus*, of the family *Araliaceae*, is abundant in Korea, China, Russia, and Japan. More than 30 *Acanthopanax* species grow in East Asia; more than 18 are known in Korea [1]. *Acanthopanax* species have been used in traditional medicine to make tonics, sedatives, and antispasmodics and to treat rheumatoid arthritis, diabetes, bacterial infections, cancer, and hypertension [2–4]. Various phytochemicals, such as lignans, steroids, and triterpenoidal saponins, have been identified in *Acanthopanax* species [5–8]. The major active component in these plants is lignan, and it has been determined that lignan plays a key role in the therapeutic effects [9, 10].

Various molecules are involved in the induction and maintenance of inflammatory responses. In addition to major cytokines, such as interleukin- (IL-) 1, IL-6, and tumor necrosis factor- (TNF-) *α*, prostaglandin and nitric oxide (NO), which is synthesized by inducible NO synthase (iNOS), are important chemical mediators of inflammation [11]. Prostaglandin is synthesized by two cyclooxygenase (COX) isoforms, COX-1 and inducible COX-2. Therefore, the inhibition and/or downregulation of these proinflammatory molecules may exert anti-inflammatory effects in arthritis [12].

Recent studies have been conducted to enhance the physiological activity of materials by fermentation using microorganisms such as fungi, yeast, lactic acid bacteria, and mushrooms. The benefits include nonsecondary pollution, a mild reaction, and the low cost of biotransformation. Microorganisms have many bioconversion elements. As a part of our previous related work [13], inhibitory activities of nitric oxide (NO) production, iNOS and COX-2, proinflammatory cytokines (IL-6 and tumor necrosis factor-*α*), and collagenase in lipopolysaccharide- (LPS-) treated RAW 264.7 macrophage cells were evaluated on the biotransformation product of *A*. *sessiliflorus*.

## 2. Materials and Methods

### 2.1. Plant Material

In April 2018, the bark of *A. sessiliflorus* was collected from Pyeongtaek, Republic of Korea, and certified by Dr. Kim (National Arboretum, Pocheon). In addition, a voucher specimen (201804-AS) was deposited at the herbarium of the College of Pharmacy, Chung-Ang University.

### 2.2. Fermentation

*Lactobacillus plantarum* subsp. *argentoratensis* was selected for this study, on the basis of a previous study [14]. *L. plantarum* subsp. *argentoratensis* was purchased from the Korean Agricultural Culture Collection (Seoul, Korea), inoculated into MRS broth, and grown at 37°C and 200 rpm for 48 h. Then, 1% (v/v) *L. plantarum* subsp. *argentoratensis* was inoculated into MRS medium with 1% (w/v) *A. sessiliflorus* extract, and fermentation was performed at 37°C and 200 rpm for 5 days. After fermentation, centrifugation was performed for 20 min at 3000 rpm, and the supernatant was evaporated to obtain the fermented *A. sessiliflorus* (FAS) extract.

### 2.3. General Experimental Procedure

Chromatography was performed using the Sephadex LH-20 column (10–24 *μ*m; GE Healthcare Bio-Science AB, Uppsala, Sweden), MCI-gel CHP 20P (75–150 *μ*m; Mitsubishi Chemical, Tokyo, Japan), and ODS-B gel (40–60 *μ*m; Daiso, Osaka, Japan). Thin-layer chromatography (TLC) was performed using a precoated silica gel 60 F_254_ plate (Merck, Darmstadt, Germany) and chloroform : methanol (15 : 1, volume ratio) and chloroform : methanol : water (80 : 20 : 2 or 6 : 4:1). The spots were detected by application of a FeCl_3_ spray and 10% H_2_SO_4_ or anisaldehyde-H_2_SO_4_, followed by heat and UV irradiation (254 nm). The chemical structures were elucidated by using several types of instrumental analysis. 1D-nuclear magnetic resonance (NMR) experiments, such as ^1^H-(600 MHz) and ^13^C-(150 MHz) NMR, were performed using an JNM-ECZ600R (JEOL, Massachusetts, USA) at the Center for Research facilities at Chung-Ang University.

### 2.4. Extraction and Isolation

The barks of *A. sessiliflorus* (3.2 kg) were extracted four times with 70% ethanol at room temperature for 3 days. The ethanol was removed under vacuum, and 143.03 g of the extract (AS) was obtained. Sixty grams of the extract was fermented by using *L. plantarum* subsp. *Argentoratensis*; the rest was stored at −20°C. The FAS extract was subjected to Sephadex LH-20 column chromatography and eluted with MeOH : H_2_O gradient (from 0 : 10 to 10 : 0); six subfractions (fr-1 to 6) were obtained. Repeated column chromatography of fr-2 using an MCI gel column with a gradient solvent system of MeOH : H_2_O (from 0 : 10 to 10 : 0) yielded **1** (acanthoside D, 119.5 mg), **2** (acanthoside B, 1.78 g), and **3** (daucosterol, 13 mg). fr-3 yielded **4** (protocatechuic acid, 693.5 mg), **5** (chlorogenic acid methyl ester, 476.6 mg), **6** (ciwujiatone, 9 mg), **7** (syringaresinol, 890.5 mg), and **8** (farnesol, 1.43 g). fr-4 yielded **9** (3,4-dicaffeoylquinic acid, 73 mg) and **10** (falcarindiol, 60.2 mg).

### 2.5. HPLC Analysis

The compounds in the FAS extract were compared with those in the AS extract by using high-pressure liquid chromatography (HPLC). The mobile phase consisted of solvent A (0.2% acetic acid in H_2_O) and B (acetonitrile (ACN); Table 1). The extracts were dissolved in 50% MeOH.

### 2.6. RAW 264.7 Cell Culture

Murine macrophage RAW 264.7 cells were purchased from the Korean Cell Line Bank (Seoul, Korea). The cells were grown in Dulbecco's modified Eagle's medium (DMEM; Welgene, Gyeongbuk, Korea) supplemented with 10% fetal bovine serum (Welgene), 100 IU/mL penicillin G, and 100 mg/mL streptomycin (Gibco BRL, Grand Island, NY, USA) at 37°C in a humidified atmosphere (approximately 5% CO_2_), and the cells were counted by using a hemocytometer.

### 2.7. Measurement of Cell Viability

The cytotoxicity was measured by the mitochondrial-dependent reduction of 3-(4,5-dimethylthiazol-2-yl)-2,5-diphenyltetrazolium-bromide (MTT; Sigma, St. Louis, MO, USA) to formazan. RAW 264.7 cells were seeded in 96-well plates at a density of 1 × 10^5^ cells/mL. After incubation for 16 h at 37°C, the cells were treated with 20 *μ*L of each sample in serum-free DMEM and incubated at 37°C in a humidified atmosphere for 24 h. The medium was then removed, and MTT solution (100 *μ*L; 0.5 mg/mL) was added to each well. After incubation for 4 h, the supernatant was aspirated. The formazan crystals in each well were dissolved in 100 *μ*L dimethyl sulfoxide, and the absorbance at 540 nm was measured by using an enzyme-linked immunosorbent assay (ELISA) plate reader (Tecan Co. Ltd., Salzburg, Australia). Relative cell viability was evaluated from the quantity of MTT converted to the insoluble formazan salt compared with distilled water as the control. The cell viability was calculated as given in the following:

(1) $$\begin{array}{c} \text{cell viability}=\frac{\text{sample OD}}{\text{control OD}}\;\times 100. \end{array}$$

### 2.8. Analysis of Inhibition of NO Production

RAW 264.7 cells (2 × 10^5^ cells/well) were seeded in 96-well plates and incubated for 16 h at 37°C in a humidified atmosphere (approximately 5% CO_2_). The cells were incubated in a serum-free medium containing the sample and 1 *μ*g/mL LPS (Sigma, St. Louis, MO, USA). After incubation for an additional 20 h, the NO content was evaluated by using the Griess assay. The supernatant was obtained, Griess reagent (0.1% naphthylethylenediamine and 1% sulphanilamide in 5% H_3_PO_4_ solution; Sigma, St. Louis, MO, USA) was added, and the absorbance at 540 nm was recorded. N^G^-monomethyl-L-arginine monoacetate salt (L-NMMA) was used as the positive control. The inhibition of NO production was calculated from equation (2). The IC_50_ value was defined as the concentration that inhibited 50% of NO production:

(2) $$\begin{array}{c} \text{inhibition rate }\left(\text{\%}\right)=100-\frac{\left(\text{sample OD}-\text{blank OD}\right)}{\left(\text{control OD}-\text{blank OD}\right)}\;\times 100. \end{array}$$

### 2.9. Determination of Proinflammatory Cytokine Levels

The concentrations of IL-6 and TNF-*α* were determined by using ELISA. RAW 264.7 cells (1 × 10^6^ cells/well) were seeded in 6-well plates and preincubated for 16 h; subsequently, the samples were treated with LPS (1 *μ*g/mL) for 24 h to induce the production of cytokines. The supernatant was evaluated using an ELISA kit (Youngin Frontier, Seoul, Korea) in accordance with the manufacturer's instructions. The cytokine concentrations were quantified by measuring the absorbance at 450 nm.

### 2.10. Western Blotting Analysis

RAW 264.7 cells (1 × 10^6^ cells/mL) were preincubated for 16 h and then treated with LPS (1 *μ*g/mL). After incubation for 24 h, the cells were harvested and washed twice with phosphate-buffered saline. The cell lysates were prepared in RIPA buffer (50 mM/L Tris–HCl [pH 7.4], 150 mM/L NaCl, 1% Triton X-100, 0.1% sodium dodecyl sulfate (SDS), and 1 mM/L EDTA; Thermo Fisher Scientific, MA, USA) for 30 min on ice. The cell lysates were centrifuged at 13,000 × *g* for 15 min at 4 °C, and 30 *μ*g of the cell lysate was separated by to 7.5% SDS-polyacrylamide gel electrophoresis. The separated proteins were transferred onto a PVDF membrane (Bio-Rad, CA, USA). Nonspecific binding to the membrane was blocked by incubation with blocking buffer (Thermo Fisher Scientific) for 60 min at room temperature. Then, the membrane was incubated with anti-mouse iNOS (1 : 500; Santa Cruz, CA, USA) and anti-mouse COX-2 (1 : 1000; BD Biosciences Pharmingen, CA, USA) overnight at 4°C. After washing, the blots were incubated with horseradish peroxidase-conjugated goat anti-mouse IgG secondary antibody (1 : 1000; Santa Cruz) for 60 min at room temperature. The bands were visualized by using the LAS-4000 luminescent image analyzer (GE Healthcare Life Sciences, NJ, USA) and ECL detection reagent (GE Healthcare Life Sciences).

### 2.11. Collagenase Assay

The collagenase assay was performed as described previously [15]. Collagenase (5 *μ*g) and 4-phenylazobenzyloxycarbonyl-L-Pro-L-Leu-Gly-L-Pro-D-Arg (0.5 mg), as a substrate of collagenase, were added to 0.1 M Tris buffer (pH 7.4) in the presence or absence of samples to a total volume of 1.7 mL. The mixture was incubated at 37°C for 30 min, and 1 mL of 25 mM citric acid solution was added to terminate the enzyme reaction. Ethyl acetate (5 mL) was added, and the absorbance of the organic layer was measured by using UV spectrophotometry at 320 nm to calculate the inhibitory activity. The collagenase inhibitory activity was calculated from equation (3), where OD control = OD of the control with collagenase-OD of the control without collagenase, and OD sample = OD of the test sample with collagenase-OD of the test sample without collagenase:

(3) $$\begin{array}{c} \text{inhibition rate }\left(\text{\%}\right)=\frac{\left(\text{OD control}-\text{OD sample}\right)}{\text{OD control}}\;\times 100. \end{array}$$

### 2.12. Statistical Analysis

All data were expressed as mean ± SD values and were evaluated by using one-way analysis of variance (ANOVA), followed by the Student–Newman–Keuls (S–N–K) test; statistical analyses were computed using Statistical Package for the Social Sciences (SPSS, Chicago, IL, USA) software package. Values were considered significantly different if the *p* value was less than 0.05. Different superscripts in the same column indicate that values are significantly distinct from the other data.

## 3. Results and Discussion

### 3.1. Isolation and Structural Identification of the Compounds

**Figure 1:**
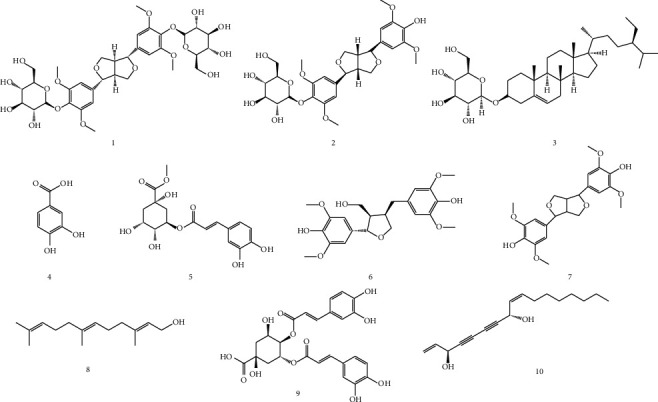
Structures of compounds **1**–**10.**

We isolated 10 compounds from FAS, namely, lignans (**1**, **2**, **6**, **7**), phenolic acids (**4**, **5**, **9**), and others (**3**, **8**, **10**). The isolated compounds were identified as acanthoside D (**1**) [16], acanthoside B (**2**) [17], daucosterol (**3**) [18], protocatechuic acid (**4**) [19], chlorogenic acid methyl ester (**5**) [20], ciwujiatone (**6**) [21], syringaresinol (**7**) [22], farnesol (**8**) [23, 24], 3,4-dicaffeoylquinic acid (**9**) [25], and falcarindiol (**10**) [26] through comparison of their spectral data with the values reported in other studies (Figure 1). Structures of the 10 compounds isolated from FAS were identified by analysis of ^1^H and ^13^C NMR spectra compared with references. ^1^H and ^13^C NMR spectra of the compounds (1–10) were included in Supplementary Materials as Figure S1–S10.

Acanthoside D (**1**): brown powder, ^1^H-NMR (600 MHz, DMSO-d6 + D_2_O): *δ* 6.61 (4H, s, H-2′, 6′, 2″, 6″), 4.84 (2H, d, *J* = 4.2 Hz, H-1‴, 1″″), 4.63 (2H, d, *J* = 3.6 Hz, H-2, 6), 4.16 (2H, dd, *J* = 9.0, 6.6 Hz. H-4*β*, 8 *β*), 3.78 (2H, dd, *J* = 9.0, 3.6 Hz, H-4*α*, 8*α*), 3.71 (12H, s, H-3′, 5′ 3″ 5″-OCH3), 3.54 (2H, m, H-6‴*β*, 6″″*β*), 3.36 (2H, dd, *J* = 6, 12.0 Hz, H-6‴*α*, 6″″*β*), 3.17-3.04 (8H, m, 2‴, 3‴, 4‴, 5‴, 2″″, 3″″, 4″″, 5″″), 3.10-3.07 (2H, m, H-1, 5); ^13^C-NMR (150 MHz, DMSO-d6 + D_2_O): *δ* 153.1(C-3′, 5′, 3″, 5″), 137.7 (C-4′, 4″), 134.2 (C-1′, 1″), 104.7 (C-2′, 6′, 2″, 6″), 103.2 (C-1‴, 1″″), 85.6 (C-2, 6), 77.6 (C-5‴, 5″″), 76.9 (C-3‴, 3″″), 74.6 (C-2‴, 2″″), 71.9 (C-4, 8), 70.3 (C-4‴, 4″″), 61.3 (C-6‴, 6″″), 56.9 (C-3′, 5′, 3″, 5″–OCH3), 54.1 (C-1, 5).

Acanthoside B (**2**): brown powder, ^1^H-NMR (600 MHz, DMSO-d6 + D_2_O): *δ* 6.61 (2H, s, H-2″, 6″), 6.55 (2H, s, H-2′, 6′), 4.83 (1H, m, H-1‴), 4.63 (1H, d, *J* = 4.8 Hz, H-2), 4.57 (1H, d, *J* = 4.2, H-6), 4.13 (2H, m, H-4*β*, 8 *β*), 3.75 (2H, dd, *J* = 5.4, 3.6 Hz, H-4*α*, 8*α*), 3.71 (6H, s, OCH3-3″,5″), 3.70 (6H, s, OCH3-3′,5′), 3.54 (1H, dd, *J* = 12.0, 1.8 Hz, H-6‴*α*), 3.60 (1H, m, H-6‴*β*), 3.16 (2H, m, H-2‴, 3‴), 3.10 (1H, m, 4‴), 3.01 (2H, m, H-1, 5), 2.99 (1H, H-5‴); ^13^C-NMR (150 MHz, DMSO-d6 + D_2_O): *δ* 153.1 (C-3′, 5′), 148.3 (C-3″, 5″), 137.8 (C-1′), 135.2 (C-4″), 134.1 (C-4′), 131.9 (C-1″), 104.6 (C-2′, 6′), 104.1 (C-2″, 6″), 103.2 (C-1‴), 85.9 (C-6), 85.6 (C-2), 77.6 (C-5‴), 76.8 (C-3‴), 74.5 (C-2‴), 71.8 (C-8), 71.7 (C-4), 70.3 (C-4‴), 60.6 (C-6‴), 56.9 (C-3′, 5′-OCH3), 56.5 (C-3″, 5″-OCH3), 54.2 (C-1), 54.1 (C-5).

Daucosterol (**3**): white powder, ^13^C-NMR (150 MHz, pyridine-d5): *δ* 140.7 (C-5), 121.8 (C-6), 102.4 (C-1′), 78.5 (C-3), 78.4 (C-3′), 77.9 (C-5′), 75.2 (C-2′), 71.5 (C-4′), 62.7 (C-6′), 56.7 (C-14), 56.1 (C-17), 50.2 (C-9), 45.9 (C-24), 42.3 (C-13), 39.8 (C-4), 39.2 (C-12), 37.3 (C-1), 36.8 (C-10), 36.2 (C-20), 34.0 (C-22), 32.0 (C-7), 31.9 (C-8), 30.1 (C-2), 29.3 (C-25), 28.4 (C-16), 26.2 (C-23), 24.4 (C-15), 23.2 (C-28), 21.1 (C-11), 19.8 (C-27), 19.3 (C-19), 19.0 (C-26), 18.9 (C-21), 12.0 (C-29), 11.8 (C-18).

Protocatechuic acid (**4**): brown needle, ^1^H-NMR (600MHz, DMSO-d6 + D_2_O): *δ* 7.29 (1H, d, *J* = 1.8 Hz, H-2), 7.25 (1H, dd, *J* = 8.4, 1.8 Hz, H-6), 6.75 (1H, d, *J* = 8.4 Hz, H-5); ^13^C-NMR (150MHz, DMSO-d6 + D_2_O): *δ* 167.9 (C-7), 150.3 (C-4), 145.2 (C-3), 122.5 (C-1), 122.1 (C-6), 117.0 (C-2), 115.6 (C-5).

Chlorogenic acid methyl ester (**5**): brown powder, ^1^H-NMR (600 MHz, DMSO-d6 + D_2_O): *δ* 7.32 (1H, d, *J* = 16.2 Hz, H-7′), 6.99 (1H, d, *J* = 2.4 Hz, H-2′), 6.92 (1H, dd, *J* = 8.4, 2.4 Hz, H-6′), 6.73 (1H, d, *J* = 8.4 Hz, H-5′), 6.07 (1H, d, *J* = 16.2 Hz, H-8′), 4.96 (1H, dd, *J* = 9.6, 5.4 Hz, H-3), 3.83 (1H, dt, *J* = 9.0, 3.6, H-5), 3.50 (3H, s, H–CH3), 2.05 (2H, m, H-2*α*, 2*β*), 1.80 (1H, m, H-6*α*), 1.71 (1H, m, H-6*β*); ^13^C-NMR (150 MHz, DMSO-d6 + D_2_O): *δ* 174.1 (C-7), 166.0 (C-9′), 148.8 (C-4′), 145.9 (C-3′), 145.7 (C-7′), 126.0 (C-1′), 122.0 (C-6′), 116.3 (C-2′), 114.9 (C-5′), 114.4 (C-8′), 73.5 (C-1), 71.5 (C-5), 69.7 (C-4), 52.4 (C–CH3), 37.6 (C-6), 35.5 (C-2).

Ciwujiatone (**6**): yellowish powder, ^1^H-NMR (600 MHz, DMSO-d6 + D_2_O): *δ* 7.25 (2H, s, H-2, 6), 6.56 (2H, s, H-2′, 6′), 4.49 (1H, d, *J* = 7.8 Hz, H-7′), 4.13 (1H, m, H-8), 4.02(2H, m, H-9), 3.78 (6H, s, H-3, 5-OCH3), 3.40 (6H, s, H-3′, 5′-OCH3), 3.46 (2H, m, H-9′), 2.43 (1H, m, H-8); ^13^C-NMR (150 MHz, DMSO-d6 + D_2_O): *δ* 198.8 (C-7), 148.2 (C-3′, 5′), 148.2 (C-3, 5), 141.9 (C-4), 135.1 (C-4′), 132.4 (C-1′), 127.4 (C-1), 106.9 (C-2, 6), 104.4 (C-2′, 6′), 83.6 (C-7′), 70.3 (C-9), 60.3 (C-9′), 56.6 (C-3), 56.6 (C-5), 56.5 (C-3′, 5′), 56.0 (C-8′), 49.0 (C-8).

Syringaresinol (**7**): light brown powder, ^1^H-NMR (600 MHz, CDCl_3_): *δ* 6.57 (4H, s, H-2″, 6″, 2‴, 6‴), 4.71 (2H, m, H-2, 6), 4.27 (2H, m, H-4*α*, 8*α*), 3.89 (2H, m, H-4*β*, 8*β*), 3.88 (12H, s, H-3″, 5″, 3‴, 5‴-OCH3), 3.08 (2H, m, H-1, 5); ^13^C-NMR (150 MHz, CDCl_3_): *δ* 147.2 (C-3′, 5′), 134.3 (C-4′), 132.2 (C-1′), 102.7 (C-2′, 6′), 86.2 (C-2, 6), 71.9 (C-4, 8), 56.5 (C-3″, 5″, 3‴, 5‴-OCH3), 54.4 (C-1, 5).

Farnesol (**8**): yellowish oil, ^1^H-NMR (600 MHz, CDCl_3_): *δ* 5.39 (1H, t, *J* = 7.2 Hz, H-2), 5.13 (2H, m, H-6, 10), 4.13 (2H, d, *J* = 7.2 Hz, H-1), 2.10-1.95 (4H, m, H-4, 5, 8, 9), 1.65 (6H, brs, H-12, 15), 1.58 (6H, brs, H-13, 14); ^13^C-NMR (150 MHz, CDCl_3_): *δ* 139.9 (C-3), 135.4 (C-7), 131.4 (C-11), 124.4 (C-10), 123.9 (C-6), 123.3 (C-2), 59.4 (C-1), 39.8 (C-8), 39.6 (C-4), 26.8 (C-9), 26.4 (C-5), 25.8 (C-12), 17.8 (C-15), 16.4 (C-13), 16.1 (C-14).

3,4-dicaffeoylquinic acid (**9**): brown powder, ^1^H-NMR (600 MHz, CD_3_OD + D_2_O): *δ* 7.57 (1H, d, *J* = 15.6 Hz, H-7′), 7.49 (1H, d, *J* = 16.2 Hz, H-7″), 7.00 (2H, m, H-2′, 2″), 6.88 (2H, dd, *J* = 8.4, 1.2 Hz, H-6′, 6″), 6.73 (1H, d, *J* = 8.4Hz, H-5′, 5″), 6.27(1H, d, *J* = 15.6 Hz, H-8′), 6.17(1H, d, *J* = 15.6 Hz, H-8″), 5.61 (1H, m, H-3), 5.09 (1H, m, H-4), 3.36 (1H, m, H-5), 2.50-2.02 (4H, m, H-2, 6); ^13^C-NMR (150 MHz, CD_3_OD + D_2_O): *δ*174.0 (C-7), 167.4 (C-9′), 167.1 (C-9″), 148.3 (C-4′, 4″), 146.5(C-7′), 146.4 (C-7″), 145.3 (C-3′), 145.3 (C-3″), 126.4 (C-1′), 126.3 (C-1″), 122.0 (C-6′), 122.0 (C-6″), 115.2 (C-5′, 5″), 113.9 (C-2′, 2″), 113.4 (C-8′), 113.4 (C-8″), 74.6 (C-1), 74.5 (C-4), 68.6 (C-5), 67.8 (C-3), 37.2 (C-2), 37.0 (C-6).

Falcarindiol (**10**): brown oil, ^1^H-NMR (600 MHz, CDCl_3_): *δ* 5.92 (1H, ddd, *J* = 16.8, 10.2, 5.4Hz, H-2), 5.60 (1H, ddt, *J* = 10.8, 7.8, 1.2 Hz, H-10), 5.50 (2H, m, H-1*α*, 9), 5.25 (1H, m, H-1*β*), 5.20 (1H, d, *J* = 7.8 Hz, H-8), 4.93 (1H, brd, *J* = 5.4Hz, H-3), 2.10 (2H, m, H-11), 1.37 (2H, m, H-12), 1.25 (8H, m, H-13, 14, 15, 16) 0.87 (3H, t-like, *J* = 7.5 Hz, H-17); ^13^C-NMR (150 MHz, CDCl_3_): *δ* 135.9 (C-2), 134.8 (C-10), 127.7 (C-9), 117.4 (C-1), 79.9 (C-7), 78.3 (C-4), 70.4 (C-5), 68.8 (C-6), 63.6 (C-3), 58.7 (C-8), 31.9 (C-15), 31.0 (C-13), 29.4 (C-14), 29.2 (C-12), 27.8 (C-11), 22.7 (C-16), 14.2 (C-17).

### 3.2. HPLC Analysis Results

**Figure 2:**
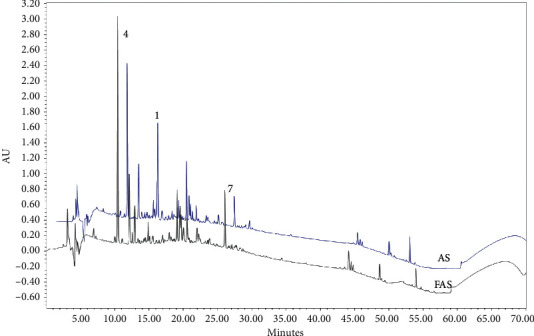
HPLC peaks for comparison of FAS and AS.

The compounds in the FAS were compared with AS by using HPLC. In this study, the remarkable differences were found in the contents of several compounds that acanthoside D (**1**) was lower and syringaresinol (**7**) and protocatechuic acid (**4**) were higher in FAS than in AS (Figure 2, Table 2).

### 3.3. Cell Viability

**Figure 3:**
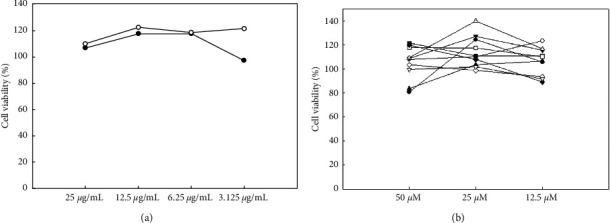
Cell viability assay. (a) Effects of AS (○) and FAS (●) extracts on the viability of RAW 264.7 cells. (b) Effects of compounds **1**–**10** on the viability of RAW 264.7 cells. **1** (●), **2** (○), **3** (▼), **4** (△), **5** (■), **6** (□), **7** (◆), **8** (◇), **9** (▲), and **10** (▽).

Prior to the biological assays, the MTT assays were performed using the extracts and each compound from the FAS extract. The extracts and compounds did not affect the cell viability (>80%) at the experimental doses (Figure 3). These results demonstrated that the concentration used in the inhibition experiments of the production of NO and cytokines and inflammatory molecules by the extracts and compounds was not cytotoxic.

To assess the anti-inflammatory activity of AS, FAS, and compounds **1**–**10** isolated from FAS, the inhibitory effects on NO production in RAW 264.7 cells were evaluated. FAS (IC_50_ = 12.31 ± 0.92 *μ*g/mL) had strong inhibitory effects on NO production compared with AS (IC_50_ = 26.56 ± 1.28 *μ*g/mL) and effects similar to those of the positive control, L-NMMA (IC_50_ = 10.16 ± 0.85 *μ*g/mL). Most of the isolated compounds had strong inhibitory effects on NO production (Table 3). Some compounds isolated from FAS, **7** (IC_50_ = 26.56 ± 1.28 *μ*M), **8** (IC_50_ = 40.63 ± 3.38 *μ*M), **9** (IC_50_ = 7.95 ± 2.03 *μ*M), and **10** (IC_50_ = 21.87 ± 1.02 *μ*M), more strongly inhibited NO production than the positive control, L-NMMA (IC_50_ = 19.68 ± 5.38 *μ*M). In particular, the aglycone form (**7**) showed better activity than the glycoside (**1**).

### 3.4. Inhibition of iNOS and COX-2 Levels

**Figure 4:**
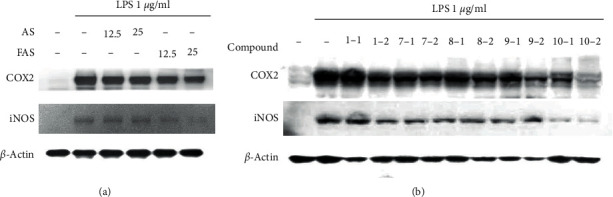
Expression of iNOS and COX-2 (western blotting). (a) Effects of AS and FAS on the expression of iNOS and COX-2 in the LPS-stimulated RAW 264.7 cells. 12.5, 12.5 *μ*g/mL; 25, 25 *μ*g/mL. (b) Effects of the selected compounds on the expression of iNOS and COX-2 in the LPS-stimulated RAW 264.7 cells. 1, 50 *μ*M; 2, 25 *μ*M.

To further characterize the mechanisms underlying the inhibition of the LPS-induced production of NO by the extracts and compounds, western blotting assays were performed. Four compounds (**7**–**10**) that showed strong inhibitory activity in the NO production assay were selected for the test of iNOS and COX-2; in addition, compound **1** was selected for comparison with its aglycone, **7**. The levels of iNOS and COX-2 were significantly increased in the LPS-stimulated cells compared with the control cells (Figure 4). Treatment with AS, FAS, and the selected compounds significantly inhibited iNOS expression in a concentration-dependent manner; similar results were obtained for COX-2. The FAS extract strongly inhibited iNOS and COX-2 levels compared with AS. Most of the compounds decreased iNOS and COX-2 production, especially **9** and **10**, and the aglycone (**7**) showed better activity than the glycoside (**1**). These results indicated that the extracts and compounds inhibited NO production through a decrease in iNOS and COX-2 expression in the LPS-stimulated RAW 264.7 cells.

### 3.5. Effects on Cytokine Production

**Figure 5:**
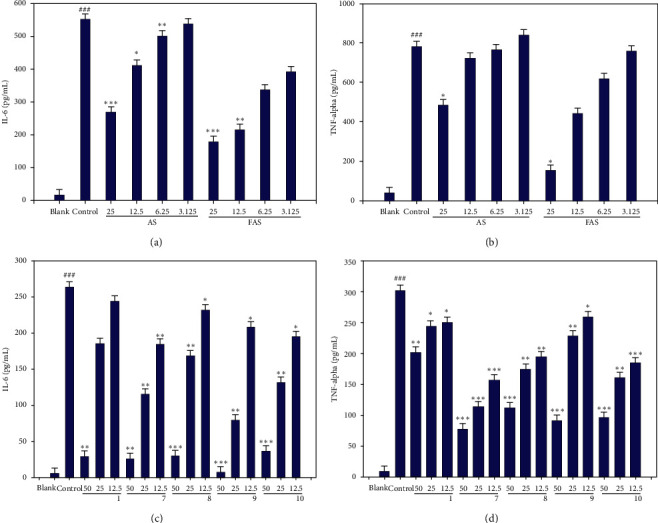
Effects of AS and FAS on IL-6 and TNF-*α* production in RAW 264.7 cells. (a) Effects of the *A. sessiliflorus* and FAS extracts on IL-6 production. (b) Effects of AS and FAS on TNF-*α* production. (c) Effects of the isolated compounds on IL-6 production. (d) Effects of the selected compounds on TNF-*α* production. The results are expressed as the mean ± SD of triplicate experiments. ^###^*p* < 0.001 when compared with the blank; ^*∗∗∗*^ *p* < 0.001, ^*∗∗*^*p* < 0.01, and ^*∗*^*p* < 0.05 when compared with the control.

The inhibition of cytokine (IL-6 and TNF-*α*) production was analyzed by using ELISA. RAW 264.7 macrophage cells were exposed to LPS, and the cytokine levels in these cells were measured for their evaluation of the inhibitory effects of AS, FAS, and the isolated compounds. In the LPS-treated group (control), IL-6 and TNF-*α* levels were detected to be 548.12 pg/mL and 777.89 pg/mL, respectively. In the AS and FAS treated groups, IL-6 and TNF-*α* levels were decreased (Figures 5(a) and 5(b)). The FAS inhibited cytokines to a greater extent than AS did. RAW 264.7 cells were exposed to LPS, and the inhibitory effects of compounds (**1** and **7–10**) on cytokine production (IL-6 and TNF-*α*) were measured. In the LPS-treated group (control), IL-6 and TNF-*α* levels were 262.07 pg/mL and 302.88 pg/mL, respectively. IL-6 and TNF-*α* levels were decreased by treatment with the selected compounds. And the **9**, **10,** and the aglycone **7** showed better activity than the glycoside (Figures 5(c) and 5(d)).

### 3.6. Effects on Collagenase Reaction

The inhibition of collagenase by AS, FAS, and the isolated compounds was evaluated. FAS (79%) more strongly inhibited collagenase when compared with AS (33%; Table 4). The effects of five compounds (**1** and **7–10**) on the collagenase reaction were measured. Compounds **9** (90.38%) and **10** (52.88%) showed potent inhibitory activities, and **1**, **7**, and **8** exhibited moderate activities. The aglycone form (**7**) (23.08%) showed better activity than the glycoside form (**1**) (15.38%).

## 4. Discussion

There are many reports that the linkage of glycosyl groups of the aglycone can alter their bioactivities [27, 28]. The variation of natural products and their bioactivities by fermentation is also reported. For example, some researchers reported that rare ginsenosides could be produced from major ginsenosides by microorganisms [27, 29]. The aglycones of ginsenosides are more easily absorbed from the small intestine than glycosides and have altered physiological actions *in vitro* and *in vivo* [30, 31]. This has been reported for not only ginsenosides, but also other compounds, including tannins and fatty acids. *L. plantarum* subsp. *argentoratensis* has tannase and can decompose tannin; thus, it has been used for the initial degradation of complex tannins [32]. The fermentation of *Vigna sinensis* L., cowpea, increased phenolic compound content and enhanced antioxidant activity [33]. In another study, microorganisms converted linoleic and linolenic acids to unsaturated hydroxy fatty acids [34]. Syringaresinol, the aglycone form of lignan (**7**), has been reported to more strongly inhibit proinflammatory molecules (prostaglandin E_2_, TNF-*α*, iNOS, and COX-2) compared with acanthoside D, the glycoside form of lignan (**1**) [3]. In addition, caffeic acid, quinic acid, and protocatechuic acid, which are common in plants, have antioxidant, antitumor, and anti-inflammatory activities [35]. In the present study, FAS showed stronger anti-inflammatory activities than AS. Moreover, the activity of syringaresinol (**7**) was better than that of acanthoside D (**1**). The HPLC analysis showed that the content of syringaresinol, the aglycone (**7**), increased during fermentation and the content of acanthoside D, the glycoside (**1**), decreased during fermentation. Protocatechuic acid (**4**) content also increased by bioconversion, and these compounds had good inhibitory effects on inflammatory-related factors. These results indicated that the variations due to bioconversion led to significant differences in the anti-inflammatory effects of the FAS and AS.

## 5. Conclusion

The biotransformation of the *A. sessiliflorus* extract via fermentation was performed. The inhibitions of the production of NO and inflammatory molecules by AS, FAS, and the isolated compounds were measured in vitro. The results showed that the content of the compounds was different in FAS and AS. The increased compounds, syringaresinol (**7**) and protocatechuic acid (**4**), showed better activity than acanthoside D (**1**). Moreover, the anti-inflammatory activities of FAS were stronger than those of AS. These results indicated that FAS and the isolated compounds may be a potential treatment for inflammatory disorders such as arthritis.

## Figures and Tables

**Table 1:** HPLC analysis conditions.

HPLC conditions			
Mobile phase	(A) 0.2% acetic acid in H_2_O	Flow rate 1 mL/min	
	(B) ACN	Injection vol. 10 *μ*L	

Gradient profile	Time (min)	A (%)	B (%)
	0	95	5
	40	40	60
	50	0	100
	55	0	100

High-pressure liquid chromatography			
Controller	Waters 2695 separation module		
Column	Hector C_18_ HPLC column (5 *μ*m, 250 × 4.6 mm)		
Detector	Waters 996 photodiode array detector		

**Table 2:** Retention time and percentage of compounds **1**, **4**, and **7** in AS and FAS.

Compound	Retention time (min)	AS (%)	FAS (%)
**1**	14.9	13.67	2.19
**4**	10.4	1.68	3.50
**7**	26.1	4.45	9.94

**Table 3:** IC_50_ values for inhibition of NO production by AS, FAS, and isolated compounds **1**–**10**.

Extract	IC_50_ (*μ*g/mL)	Compound	IC_50_ (*μ*M)
AS	26.56 ± 1.28^b^	**1**	57.71 ± 2.33^c^
FAS	12.37 ± 0.92^a^	**2**	>100^e^
l-NMMA	10.16 ± 0.85^a^	**3**	>100^d^
		**4**	40.63 ± 3. 38^b,c^
		**5**	45.80 ± 8.28^b,c^
		**6**	>100^d^
		**7**	26.16 ± 1. 37^a,b^
		**8**	37.35 ± 7.97^b,c^
		**9**	7.95 ± 2.03^a^
		**10**	21.87 ± 1.02^a,b^
		l-NMMA	19.68 ± 5.38^a,b^

Values are expressed as the mean ± SD of triplicate experiments. Values with different superscripts (a–e) in the same columns are significantly different (*p* < 0.05).

**Table 4:** Effects of AS, FAS, and the selected compounds on the collagenase reaction.

Extract (25 *μ*g/mL)	Inhibition (% of control)	Extract (50 *μ*M)	Inhibition (% of control)
AS	33.00	**1**	15.38
FAS	79.00	**7**	23.08
		**8**	22.12
		**9**	90.38
		**10**	52.88

## Data Availability

The data used to support the findings of this study are included within the article.

## Supplementary Materials

Supplementary Materials Supplementary materials file includes NMR spectra of the 10 compound isolated from FAS. Figure S1-1: ^1^H-NMR spectrum of compound **1** (600 MHz, DMSO + D_2_O); Figure S1-2: ^13^C-NMR spectrum of compound **1** (150 MHz, DMSO + D_2_O); Figure S2-1: ^1^H-NMR spectrum of compound **2** (600 MHz, DMSO + D_2_O); Figure S2-2: ^13^C-NMR spectrum of compound **2** (150 MHz, DMSO + D_2_O); Figure S3-1: ^1^H-NMR spectrum of compound **3** (600 MHz, pyridine-d5); Figure S3-2: ^13^C-NMR spectrum of compound **3** (150 MHz, pyridine-d5); Figure S4-1: ^1^H-NMR spectrum of compound **4** (600 MHz, DMSO + D_2_O); Figure S4-2: ^13^C-NMR spectrum of compound **4** (150 MHz, DMSO + D_2_O); Figure S5-1: ^1^H-NMR spectrum of compound **5** (600 MHz, DMSO + D_2_O); Figure S5-2: ^13^C-NMR spectrum of compound **5** (150 MHz, DMSO + D_2_O); Figure S6-1: ^1^H-NMR spectrum of compound **6** (600 MHz, DMSO + D_2_O); Figure S6-2: ^13^C-NMR spectrum of compound **6** (150 MHz, DMSO + D_2_O); Figure S7-1: ^1^H-NMR spectrum of compound **7** (600 MHz, CDCl_3_); Figure S7-2: ^13^C-NMR spectrum of compound **7** (150 MHz, CDCl_3_); Figure S8-1: ^1^H-NMR spectrum of compound **8** (600 MHz, CDCl_3_); Figure S8-2: ^13^C-NMR spectrum of compound **8** (150 MHz, CDCl_3_); Figure S9-1: ^1^H-NMR spectrum of compound **9** (600 MHz, CD_3_OD + D_2_O); Figure S9-2: ^13^C-NMR spectrum of compound **9** (150 MHz, CD_3_OD + D_2_O); Figure S10-1: ^1^H-NMR spectrum of compound **10** (600 MHz, CDCl_3_); Figure S10-2: ^13^C-NMR spectrum of compound **10** (150 MHz, CDCl_3_).

## References

[1] Yook C. S., Lee D. H., Seo Y. K. (1976). A new forma of *acanthopanx* species (I). *Korean Journal of Anesthesiology*.

[2] Davydov M., Krikorian A. D. (2000). Eleutherococcus senticosus (rupr. & maxim.) maxim. (Araliaceae) as an adaptogen: a closer look. *Journal of Ethnopharmacology*.

[3] Jung H. J., Park H. J., Kim R. G. (2003). In vivo anti-inflammatory and antinociceptive effects of liriodendrin isolated from the stem bark of *Acanthopanax senticosus*. *Planta Medica*.

[4] Song L., Wu Y., Hu L. (1999). *Zhong Hua Ben Cao*.

[5] Yoshizumi K., Hirano K., Ando H. (2006). Lupane-type saponins from leaves ofAcanthopanax sessiliflorusand their inhibitory activity on pancreatic lipase. *Journal of Agricultural and Food Chemistry*.

[6] Lee D.-Y., Seo K.-H., Jeong R.-H. (2012). Anti-inflammatory lignans from the fruits of *Acanthopanax sessiliflorus*. *Molecules*.

[7] Lee D.-Y., Seo K.-H., Lee D.-S. (2012). Bioactive 3,4-seco-triterpenoids from the fruits of *Acanthopanax sessiliflorus*. *Journal of Natural Products*.

[8] Song Y., Deng Y., Huang D., Wen J., Liu Z., Li F. (2012). LC-MS/MS determination and pharmacokinetic study of four lignan components in rat plasma after oral administration of Acanthopanax sessiliflorus extract. *Journal of Ethnopharmacology*.

[9] Lee S., Son D., Ryu J. (2004). Anti-oxidant activities ofacanthopanax senticosus stems and their lignan components. *Archives of Pharmacal Research*.

[10] Lyu S.-Y., Park W.-B. (2010). Modulation of IL-12 and IFN-*γ* secretions by eleutheroside E, tortoside A, and syringaresinol from Acanthopanax koreanum nakai. *Biomolecules and Therapeutics*.

[11] Chettibi S., Ferguson M., Gallin J., Snyderman R., Gallin J. I., Williams S. R., Lipincott W. (1999). *Inflammation: Basic Principles and Clinical Correlates*.

[12] Goldring M. B. (2000). Osteoarthritis and cartilage: the role of cytokines. *Current Rheumatology Reports*.

[13] Yin J., Heo J., Hwang Y., Le T., Lee M. (2016). Inhibitory activities of phenolic compounds isolated from adina rubella leaves against 5*α*-reductase associated with benign prostatic hypertrophy. *Molecules*.

[14] Kim H. G., Kim K. Y., Cha C. J. (2007). Screening for ginseng-fermenting microorganisms capable of biotransforming ginsenosides. *The Microbiological Society of Korea*.

[15] Sawabe Y., Yamasaki K., Iwagami S., Kajimura K., Nakagomi K. (1998). Inhibitory effects of natural medicines on the enzymes related to the skin. *Yakugaku Zasshi*.

[16] Park H. B., Lee K. H., Kim K. H. (2009). Lignans from the roots of *Berberis amurensis*. *Natural Product Sciences*.

[17] Lami N., Kadota S., Kikuchi T., Momose Y. (1991). Constituents of the roots of *Boerhaavia diffusa* L. III. Identification of Ca^2+^ channel antagonistic compound from the methanol extract. *Chemical & Pharmaceutical Bulletin*.

[18] Lee M., Lee D. G., Lee K. H. (2013). Isolation and identification of phytochemical constituents from the fruits of *Acanthopanax senticosus*. *African Journal of Pharmacy and Pharmacology*.

[19] An L. J., Guan S., Shi G. F., Bao Y. M., Duan Y. L., Jiang B. (2006). Protocatechuic acid from *Alpinia oxyphylla* against MPP+ induced neurotoxicity in PC12 cells. *Food and Chemical Toxicology*.

[20] Jung H. A., Park J. C., Chung H. Y., Kim J., Choi J. S. (1999). Antioxidant flavonoids and chlorogenic acid from the leaves of *Eriobotrya japonica*. *Archives of Pharmacal Research*.

[21] Wu L., Zheng J., Jiang B. (1999). Chemical constituents of the stems and leaves of *Acanthopanax senticosus* (rupr, ET maxim.) harms. *Acta Pharmaceutica Sinica*.

[22] Deyama T., Ikawa T., Nishibe S. (1985). The constituents of *Eucommia ulmoides* Oliv. II. Isolation and structures of three new lignan glycosides. *Chemical & Pharmaceutical Bulletin*.

[23] Bradesi P., Tomi F., Casanova J. (1995). Carbon-13 NMR study of farnesol, farnesyl acetate and farnesal stereoisomers: chemical shift assignment using lanthanide induced shifts. *Canadian Journal of Applied Spectroscopy*.

[24] Miyazawa M., Nankai H., Kameoka H. (1996). Biotransformation of acyclic terpenoid (2E,6E)-farnesol by plant pathogenic fungus Glomerella cingulata. *Phytochemistry*.

[25] Basnet P., Matsushige K., Hase K., Kadota S., Namba T. (1996). Four di-O-caffeoyl quinic acid derivatives from propolis. Potent hepatoprotective activity in experimental liver injury models. *Biological & Pharmaceutical Bulletin*.

[26] Fujioka T., Furumi K., Fujii H. (1999). Antiproliferative constituents from umbelliferae plants. V. A new furanocoumarin and falcarindiol furanocoumarin ethers from the root of *Angelica japonica*. *Chemical & Pharmaceutical Bulletin*.

[27] Wu Y.-Y., Cui Y.-N., Zhang T.-Y. (2018). Transformation of ginsenoside Rh4 and its aglycone from the total saponins of stems and leaves of *Panax ginseng* by *Aspergillus tubingensis*. *Phytochemistry Letters*.

[28] Park J. S., Rho H. S., Kim D. H., Chang I. S. (2006). Enzymatic preparation of kaempferol from green tea seed and its antioxidant activity. *Journal of Agricultural and Food Chemistry*.

[29] Fu Y., Yin Z. H., Yin C. Y. (201). Biotransformation of ginsenoside Rb1 to ginsenoside Rg3 by endophytic bacterium *Burkholderia* sp. GE 17‐7 isolated from *Panax ginseng*. *Journal of Applied Microbiology*.

[30] Hu C., Song G., Zhang B. (2012). Intestinal metabolite compound K of panaxoside inhibits the growth of gastric carcinoma by augmenting apoptosis via Bid-mediated mitochondrial pathway. *Journal of Cellular and Molecular Medicine*.

[31] Joh E.-H., Lee I.-A., Jung I.-H., Kim D.-H. (2011). Ginsenoside Rb1 and its metabolite compound K inhibit IRAK-1 activation-the key step of inflammation. *Biochemical Pharmacology*.

[32] Jiménez N., Esteban-Torres M., Mancheño J. M., de las Rivas B., Muñoz R. (2014). Tannin degradation by a novel tannase enzyme present in some *Lactobacillus plantarum* strains. *Applied and Environmental Microbiology*.

[33] Dueñas M., Fernández D., Hernández T., Estrella I., Muñoz R. (2005). Bioactive phenolic compounds of cowpeas (Vigna sinensisL). Modifications by fermentation with natural microflora and with *Lactobacillus plantarum* ATCC 14917. *Journal of the Science of Food and Agriculture*.

[34] Koritala S., Bagby M. O. (1992). Microbial conversion of linoleic and linolenic acids to unsaturated hydroxy fatty acids. *Journal of the American Oil Chemists’ Society*.

[35] Liu C.-L., Wang J.-M., Chu C.-Y., Cheng M.-T., Tseng T.-H. (2002). In vivo protective effect of protocatechuic acid on tert-butyl hydroperoxide-induced rat hepatotoxicity. *Food and Chemical Toxicology*.

