# Supplementary material for: Anti-Inflammatory Effects of Fermented Bark of Acanthopanax sessiliflorus and Its Isolated Compounds on Lipopolysaccharide-Treated RAW 264.7 Macrophage Cells
Source: Evid Based Complement Alternat Med. 2020 Jul 21;2020:6749425. doi: 10.1155/2020/6749425 (PMC7391118; doi:10.1155/2020/6749425)
Supplement: Supplementary Materials — file includes NMR spectra of the 10 compound isolated from FAS. Figure S1-1: 1H-NMR spectrum of compound 1 (600 MHz, DMSO + D2O); Figure S1-2: 13C-NMR spectrum of compound 1 (150 MHz, DMSO + D2O); Figure S2-1: 1H-NMR spectrum of compound 2 (600 MHz, DMSO + D2O); Figure S2-2: 13C-NMR spectrum of compound 2 (150 MHz, DMSO + D2O); Figure S3-1: 1H-NMR spectrum of compound 3 (600 MHz, pyridine-d5); Figure S3-2: 13C-NMR spectrum of compound 3 (150 MHz, pyridine-d5); Figure S4-1: 1H-NMR spectrum of compound 4 (600 MHz, DMSO + D2O); Figure S4-2: 13C-NMR spectrum of compound 4 (150 MHz, DMSO + D2O); Figure S5-1: 1H-NMR spectrum of compound 5 (600 MHz, DMSO + D2O); Figure S5-2: 13C-NMR spectrum of compound 5 (150 MHz, DMSO + D2O); Figure S6-1: 1H-NMR spectrum of compound 6 (600 MHz, DMSO + D2O); Figure S6-2: 13C-NMR spectrum of compound 6 (150 MHz, DMSO + D2O); Figure S7-1: 1H-NMR spectrum of compound 7 (600 MHz, CDCl3); Figure S7-2: 13C-NMR spectrum of compound 7 (150 MHz, CDCl3); Figure S8-1: 1H-NMR spectrum of compound 8 (600 MHz, CDCl3); Figure S8-2: 13C-NMR spectrum of compound 8 (150 MHz, CDCl3); Figure S9-1: 1H-NMR spectrum of compound 9 (600 MHz, CD3OD + D2O); Figure S9-2: 13C-NMR spectrum of compound 9 (150 MHz, CD3OD + D2O); Figure S10-1: 1H-NMR spectrum of compound 10 (600 MHz, CDCl3); Figure S10-2: 13C-NMR spectrum of compound 10 (150 MHz, CDCl3). [file 6749425.f1.pdf]

Evidence-Based Complementary and Alternative Medicine

Anti-inflammatory effects of fermented bark of *Acanthopanax sessiliflorus* and its isolated compounds on lipopolysaccharide-treated RAW 264.7 macrophage cells

Min Ji Kim[1], Hye Soo Wang[1], and Min Won Lee[1]

[1] Laboratory of Pharmacognosy and Natural Product derived Medicine, College of Pharmacy, Chung-Ang University, Seoul 06974, Korea

Correspondence should be addressed to Min Won Lee; mwlee@cau.ac.kr

Supplementary Materials

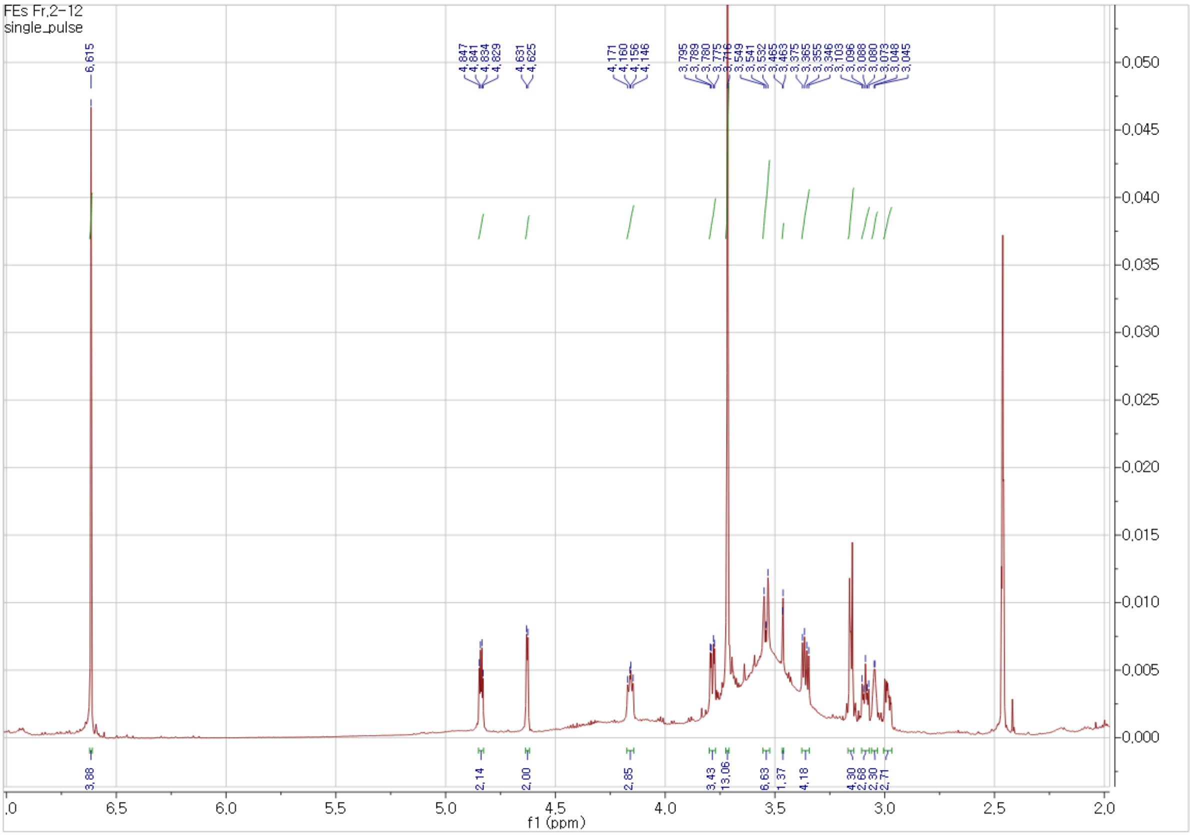

Figure S1-1: ^{1}H-NMR spectrum of compound **1** (600 MHz, DMSO+D_2O)

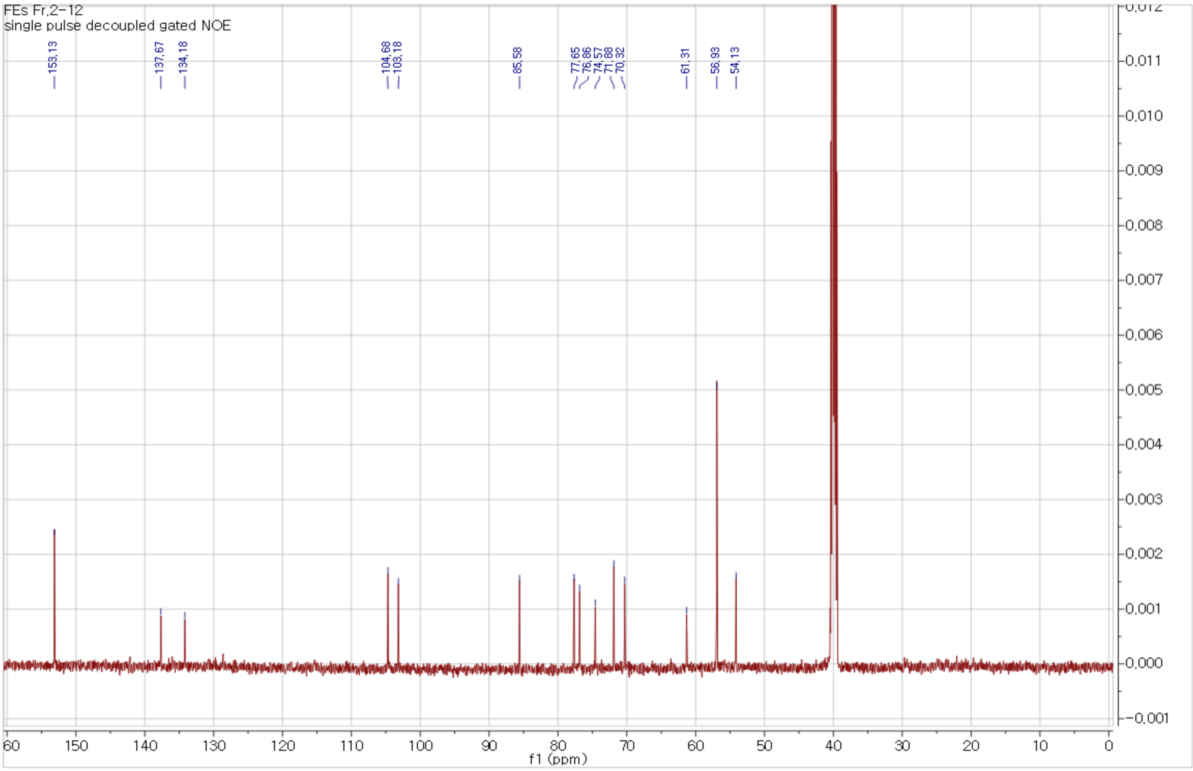

Figure S1-2: ^{13}C-NMR spectrum of compound **1** (150 MHz, DMSO+D_2O)

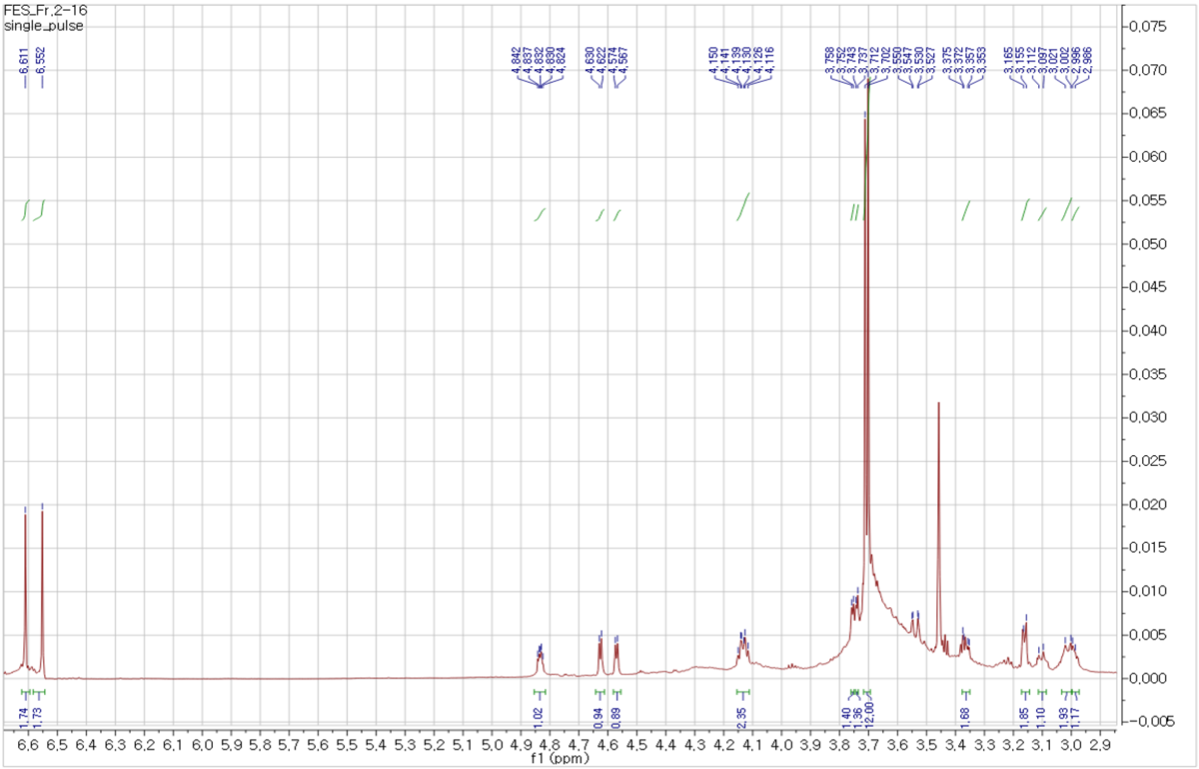

Figure S2-1: ^{1}H-NMR spectrum of compound **2** (600 MHz, DMSO+D_2O)

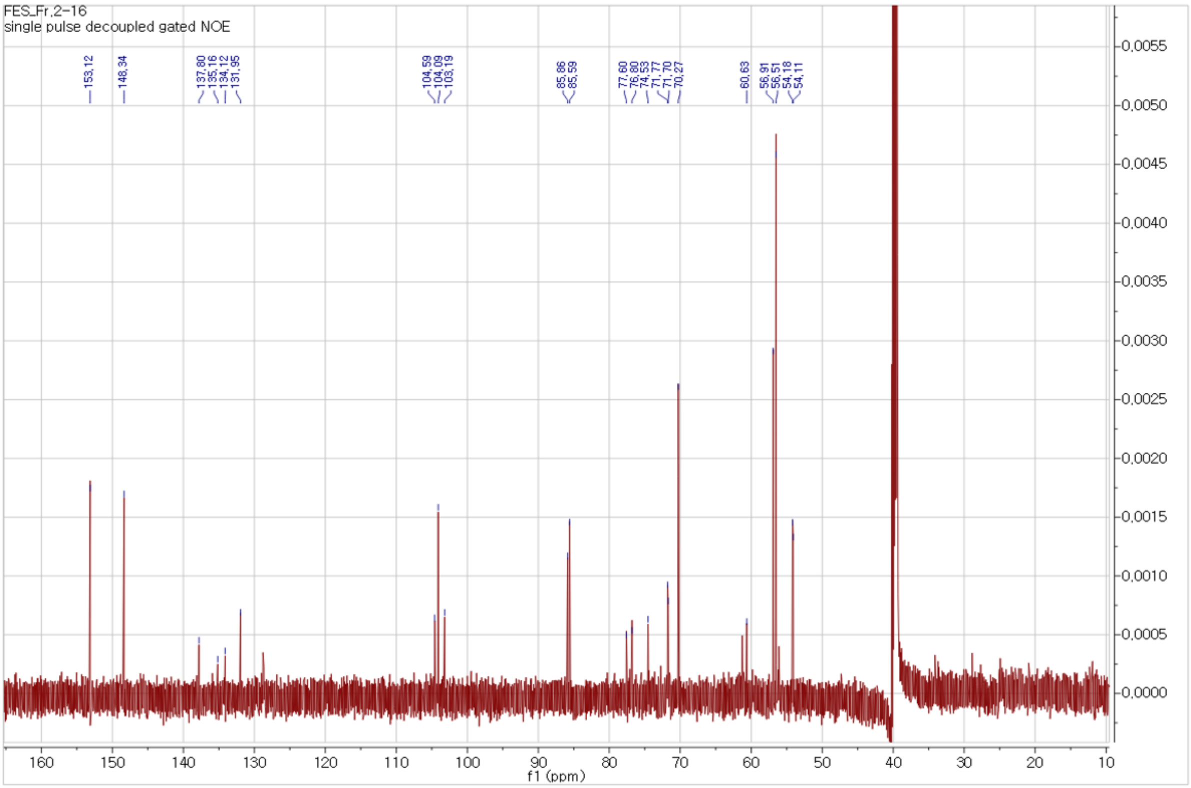

Figure S2-2: ^{13}C-NMR spectrum of compound **2** (150 MHz, DMSO+D_2O)

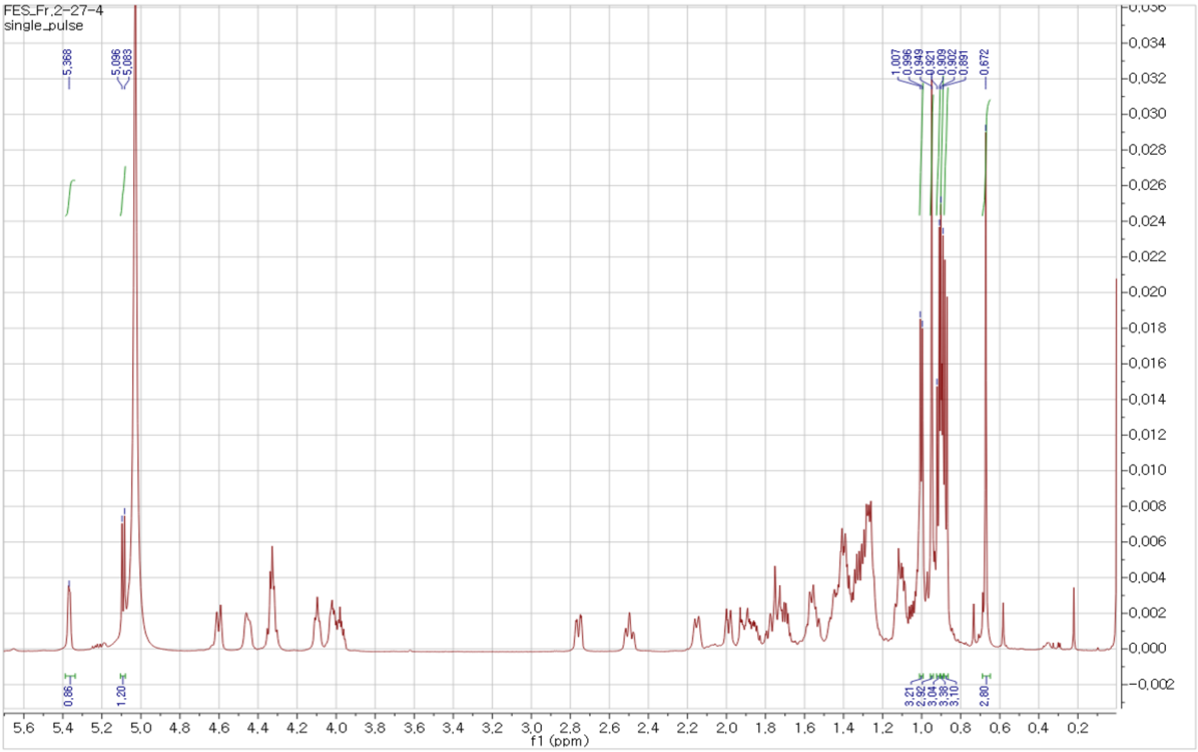

Figure S3-1: ^{1}H-NMR spectrum of compound **3** (600 MHz, pyridine-d5)

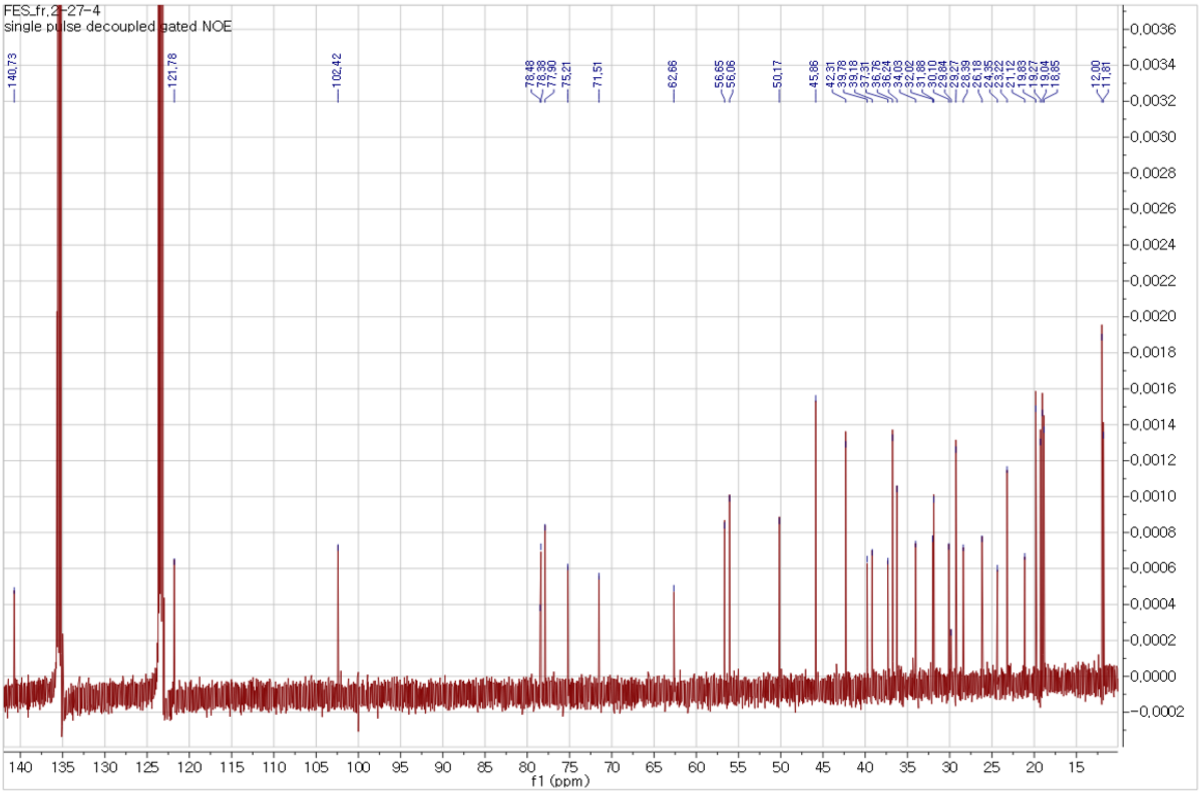

24
25 Figure S3-2: ^{13}C-NMR spectrum of compound **3** (150 MHz, pyridine-d5)
26

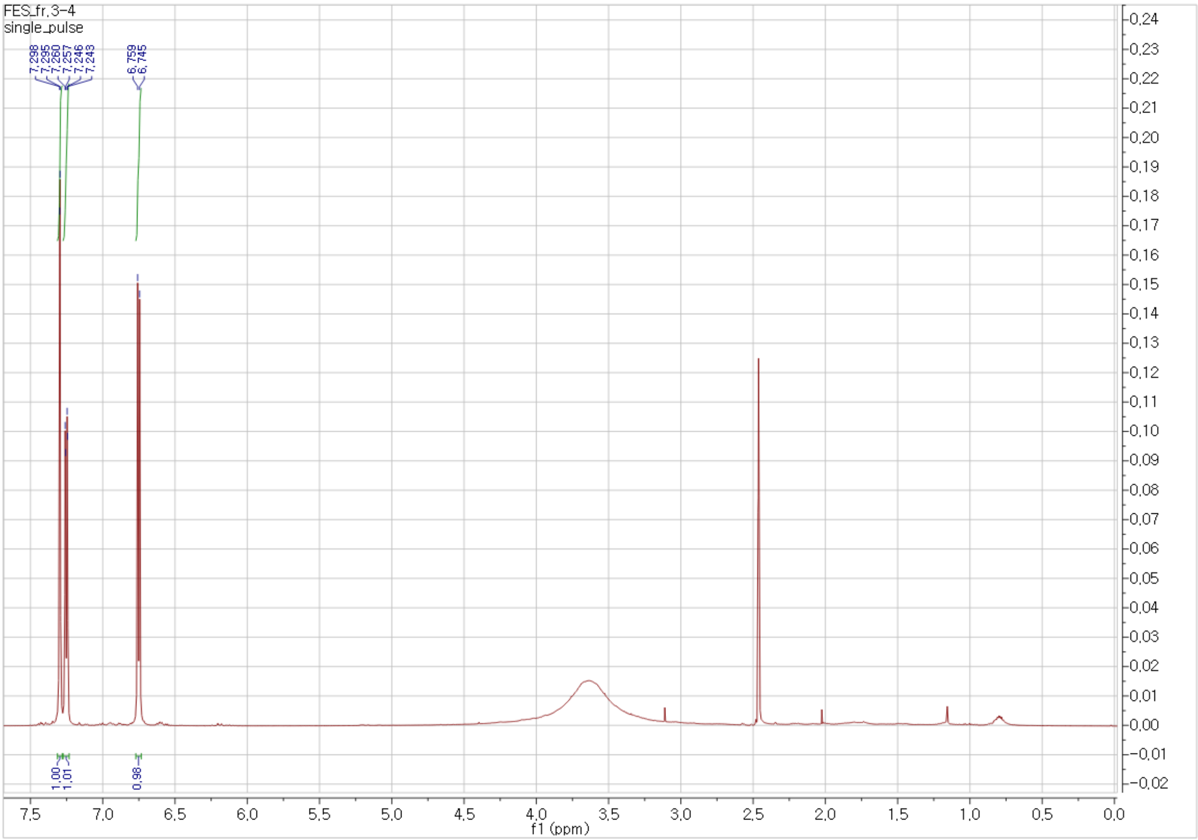

Figure S4-1: ^{1}H-NMR spectrum of compound **4** (600 MHz, DMSO+D_2O)

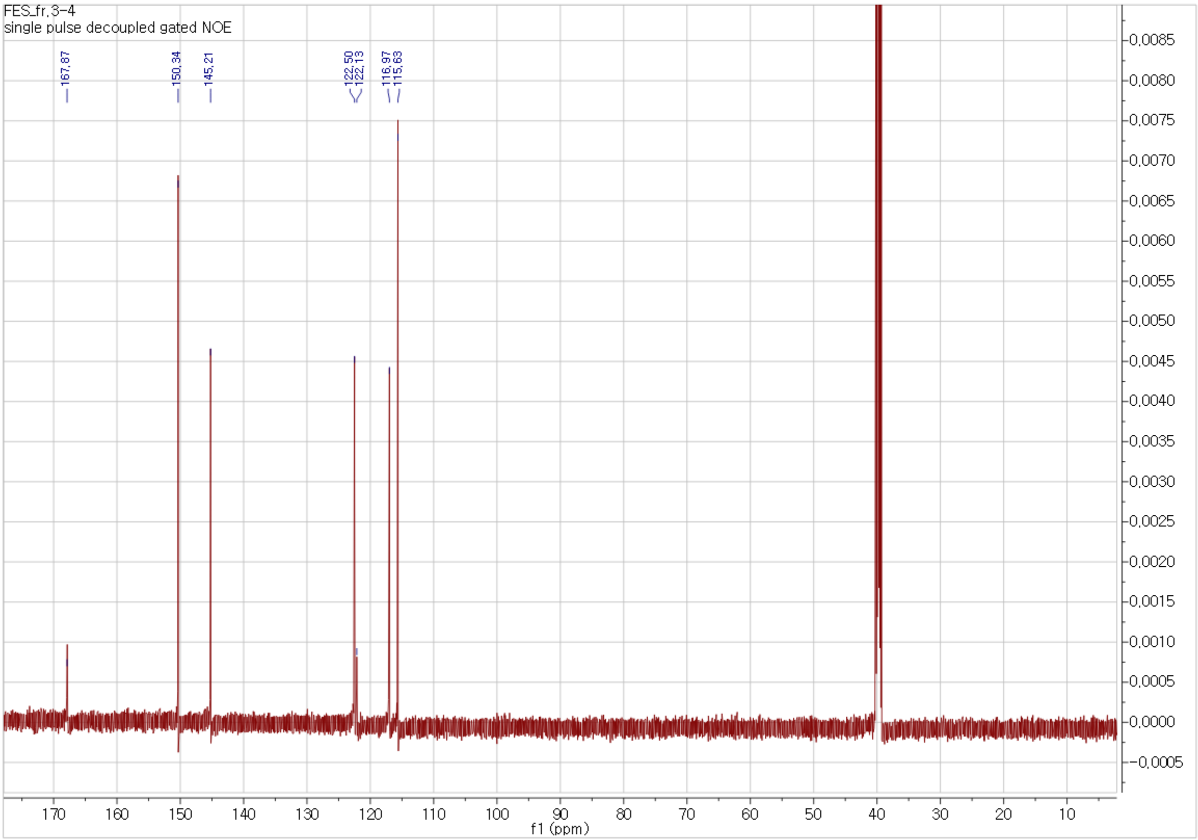

Figure S4-2: ^{13}C-NMR spectrum of compound **4** (150 MHz, DMSO+D_2O)

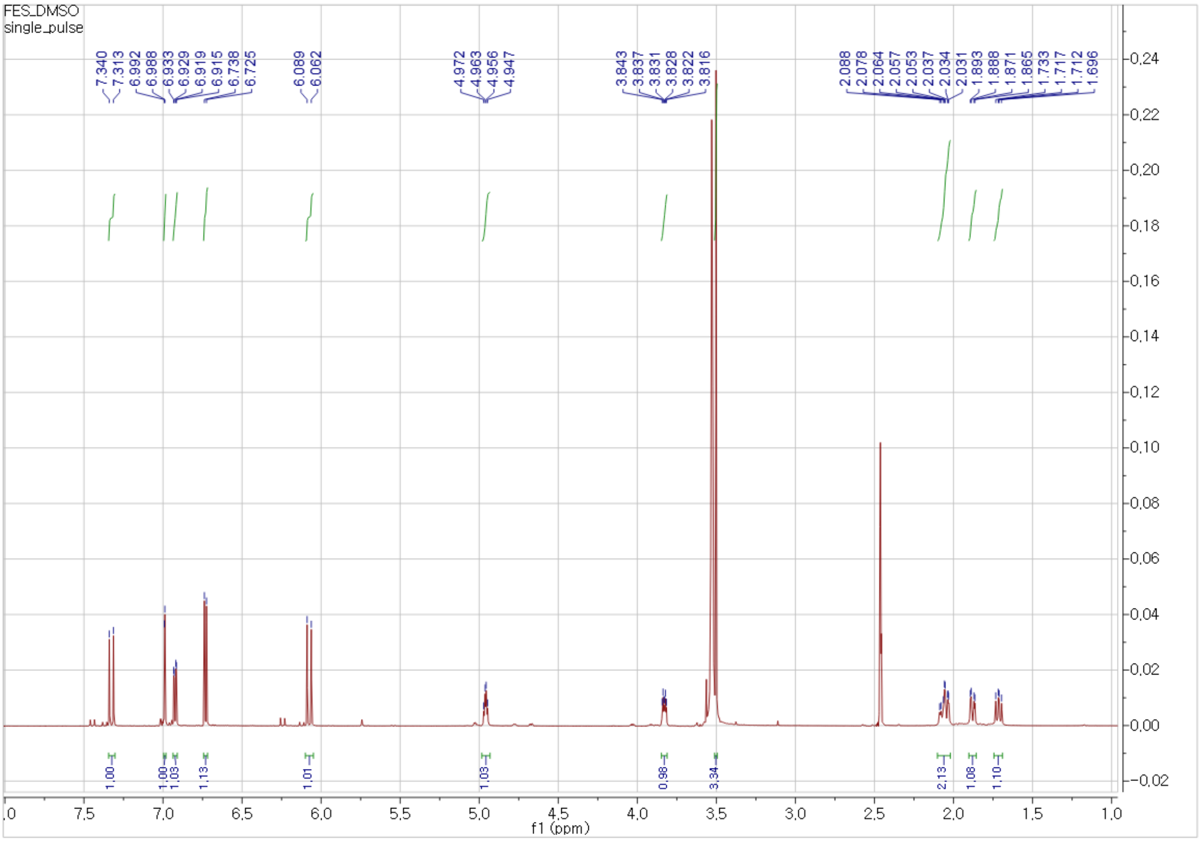

Figure S5-1: ^{1}H-NMR spectrum of compound **5** (600 MHz, DMSO+D_2O)

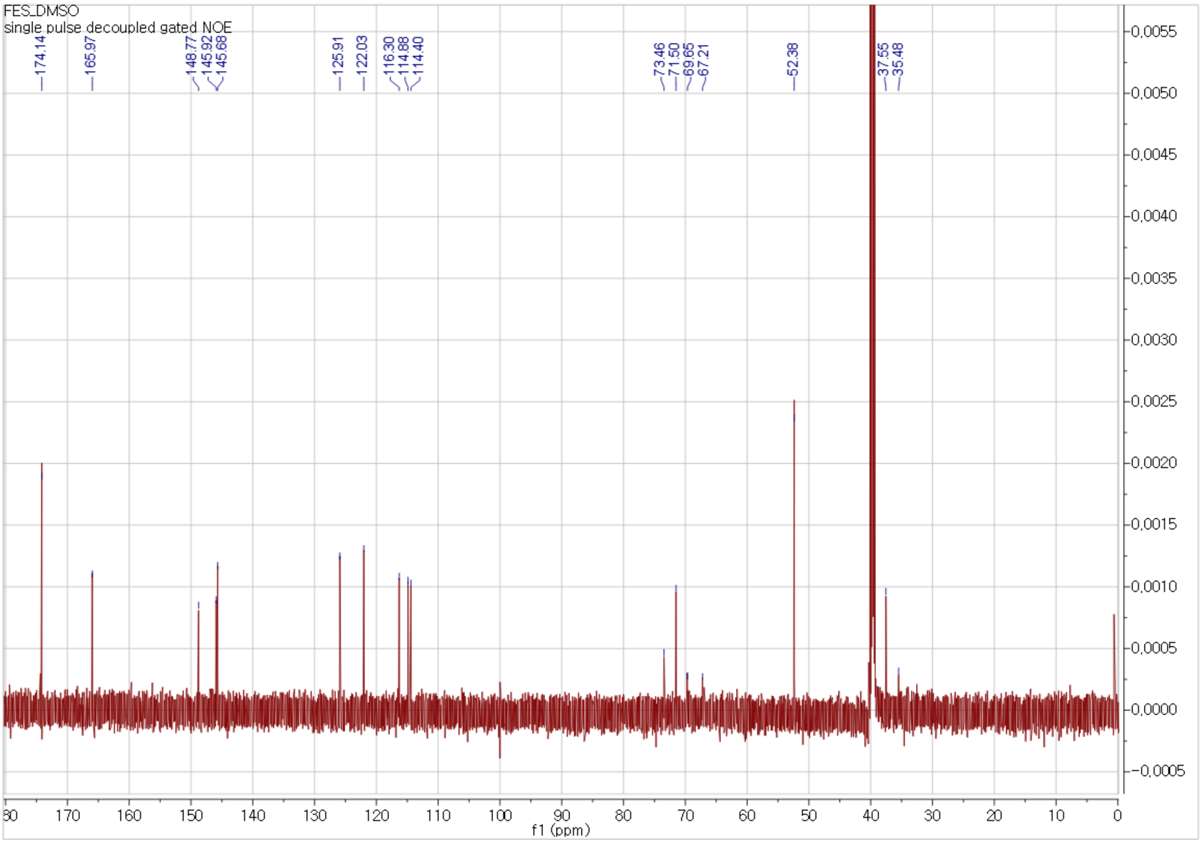

Figure S5-2: ^{1}H-NMR spectrum of compound **5** (150 MHz, DMSO+D_2O)

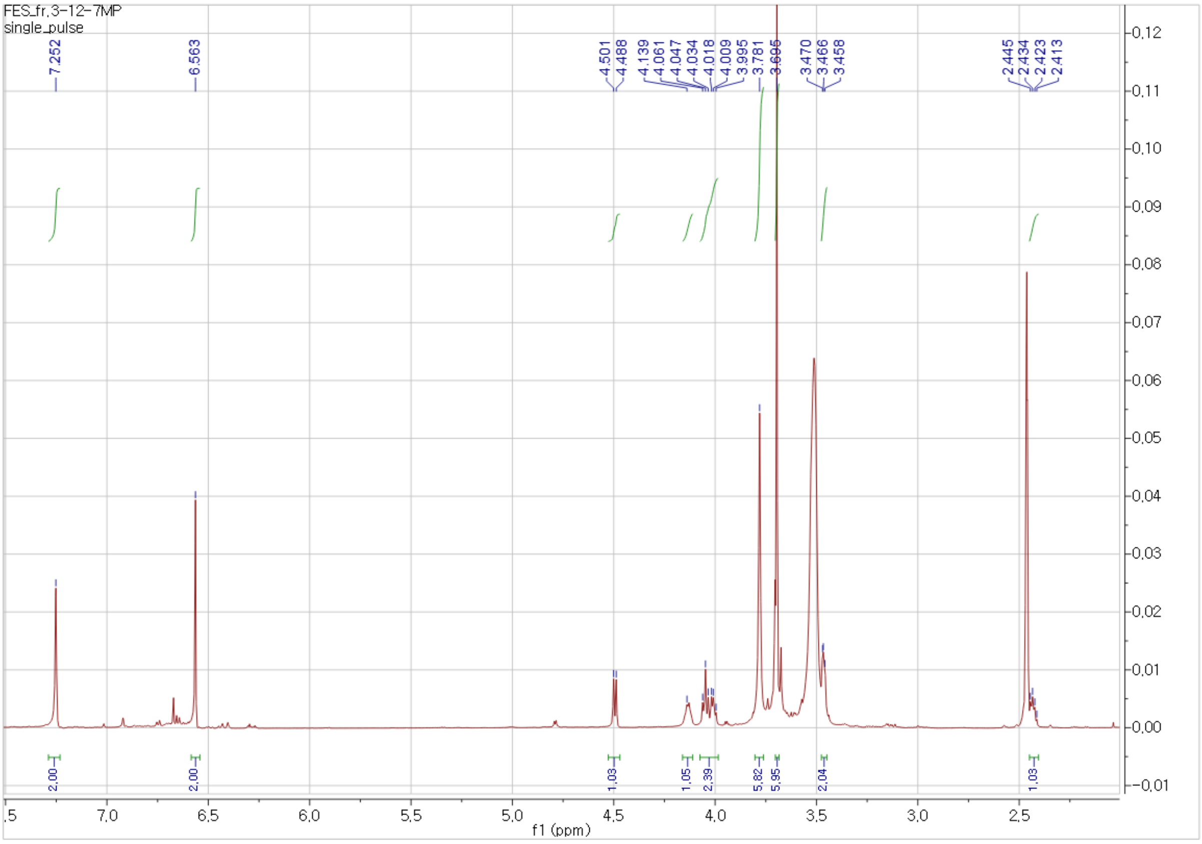

Figure S6-1: ^{1}H-NMR spectrum of compound **6** (600 MHz, DMSO+D_2O)

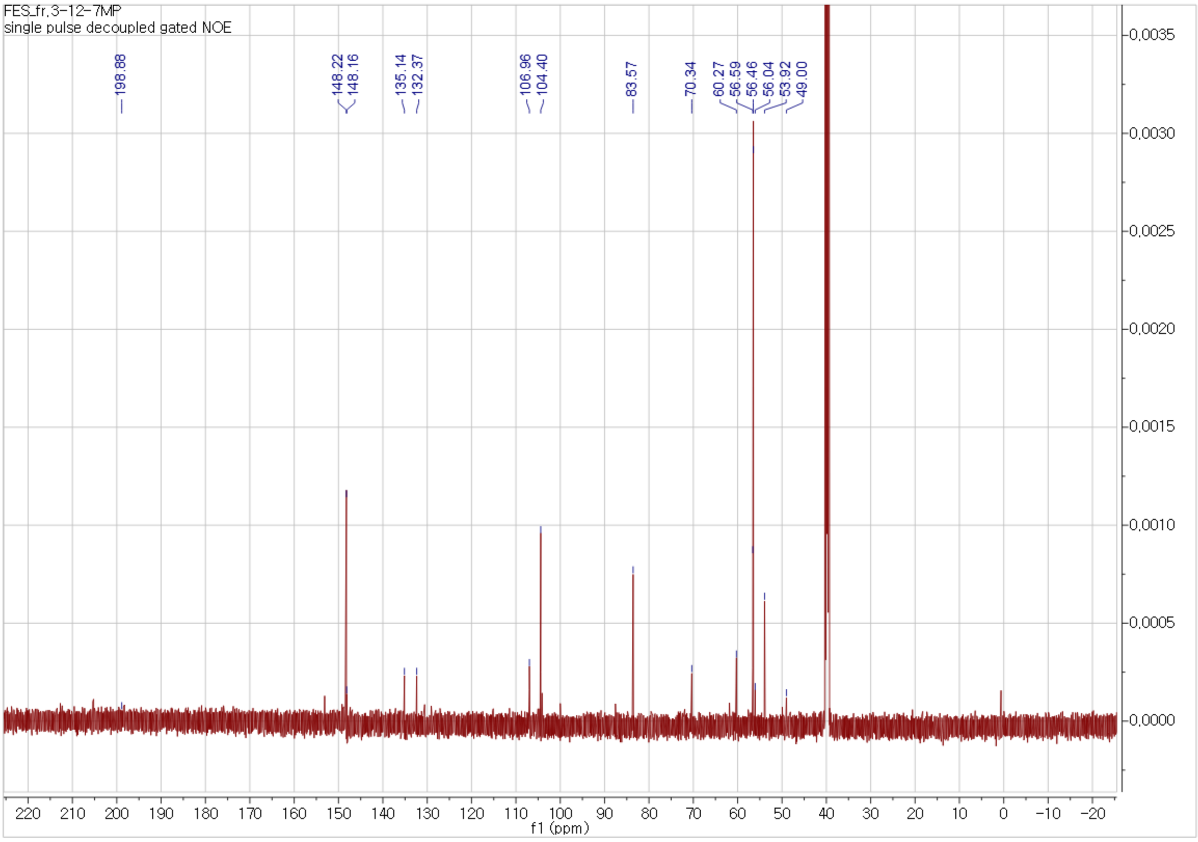

Figure S6-2: ^{1}H-NMR spectrum of compound **6** (150 MHz, DMSO+D_2O)

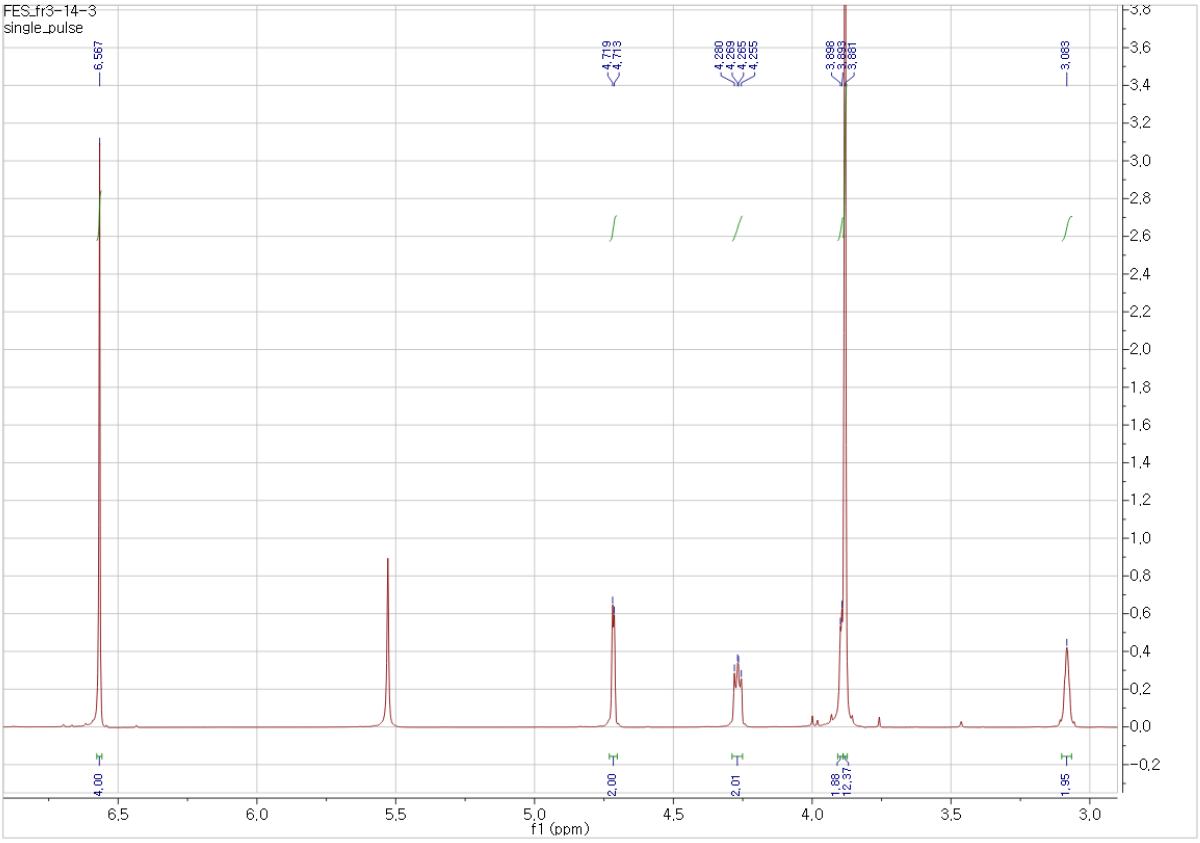

Figure S7-1: ^{1}H-NMR spectrum of compound **7** (600 MHz, $CDCl_3$)

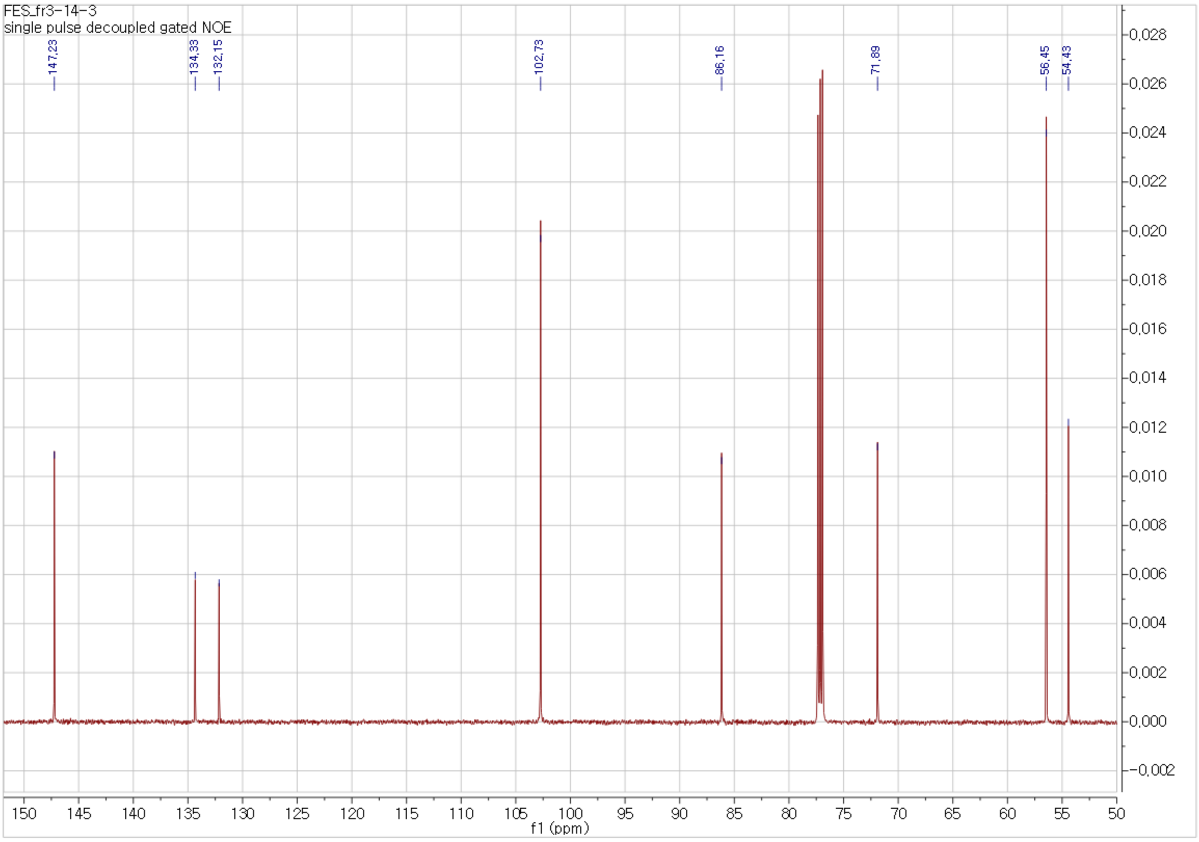

Figure S7-2: ^{13}C-NMR spectrum of compound **7** (600 MHz, $CDCl_3$)

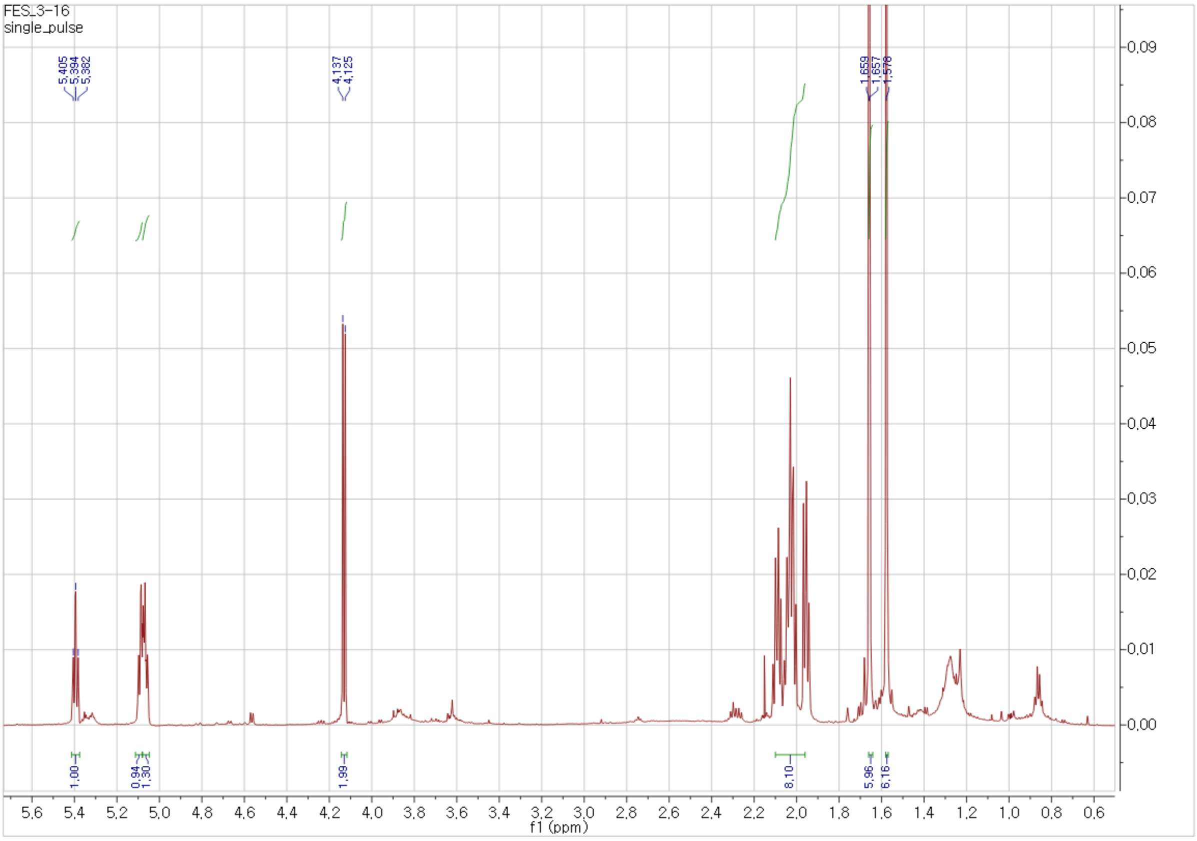

Figure S8-1: ^{1}H-NMR spectrum of compound **8** (600 MHz, $CDCl_3$)

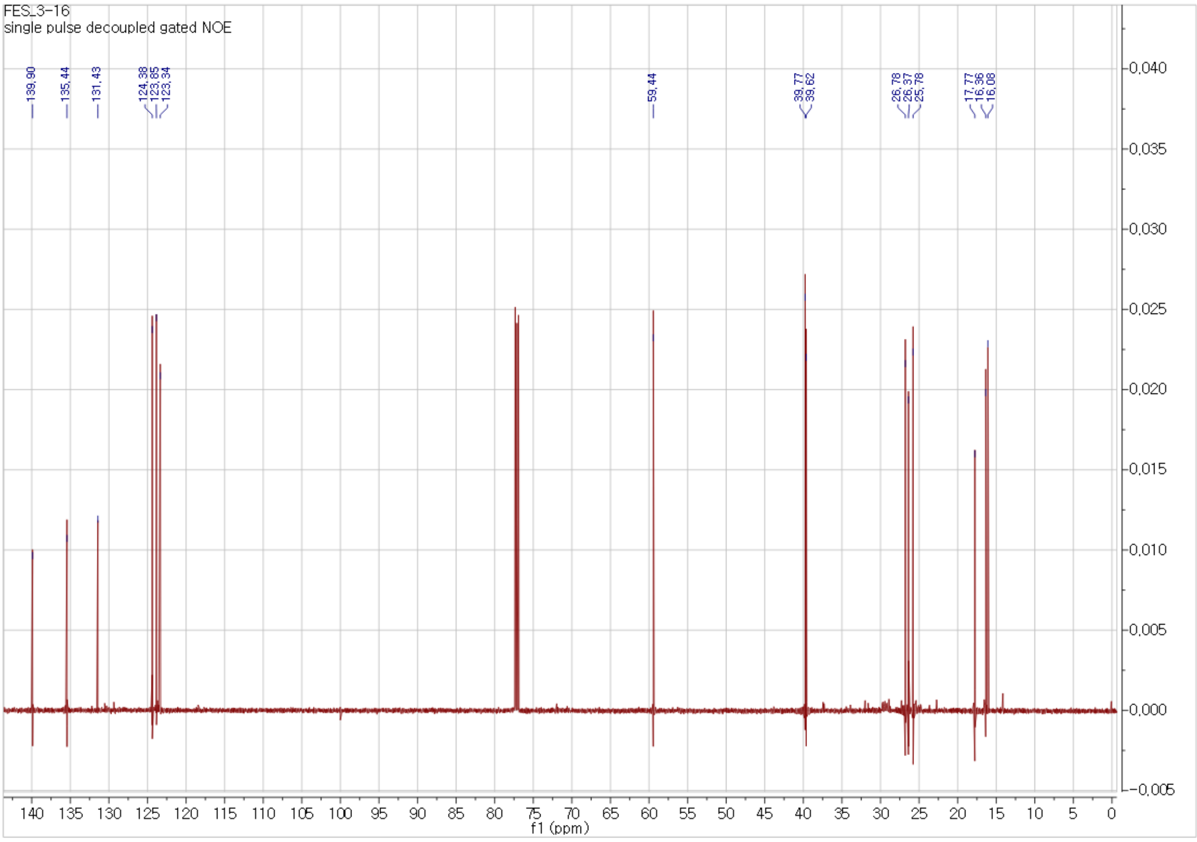

Figure S8-2: ^{13}C-NMR spectrum of compound **8** (150 MHz, $CDCl_3$)

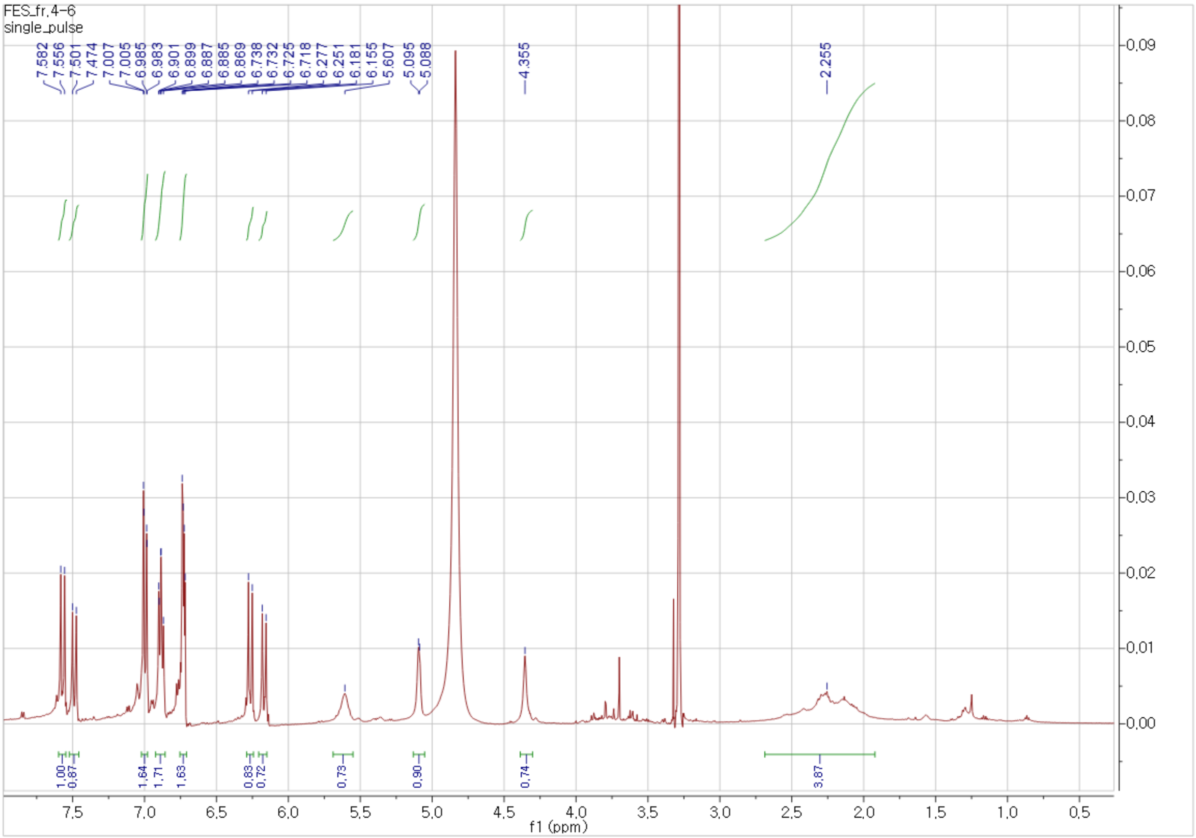

Figure S9-1: ^{1}H-NMR spectrum of compound **9** (600 MHz, CD_3OD+D_2O)

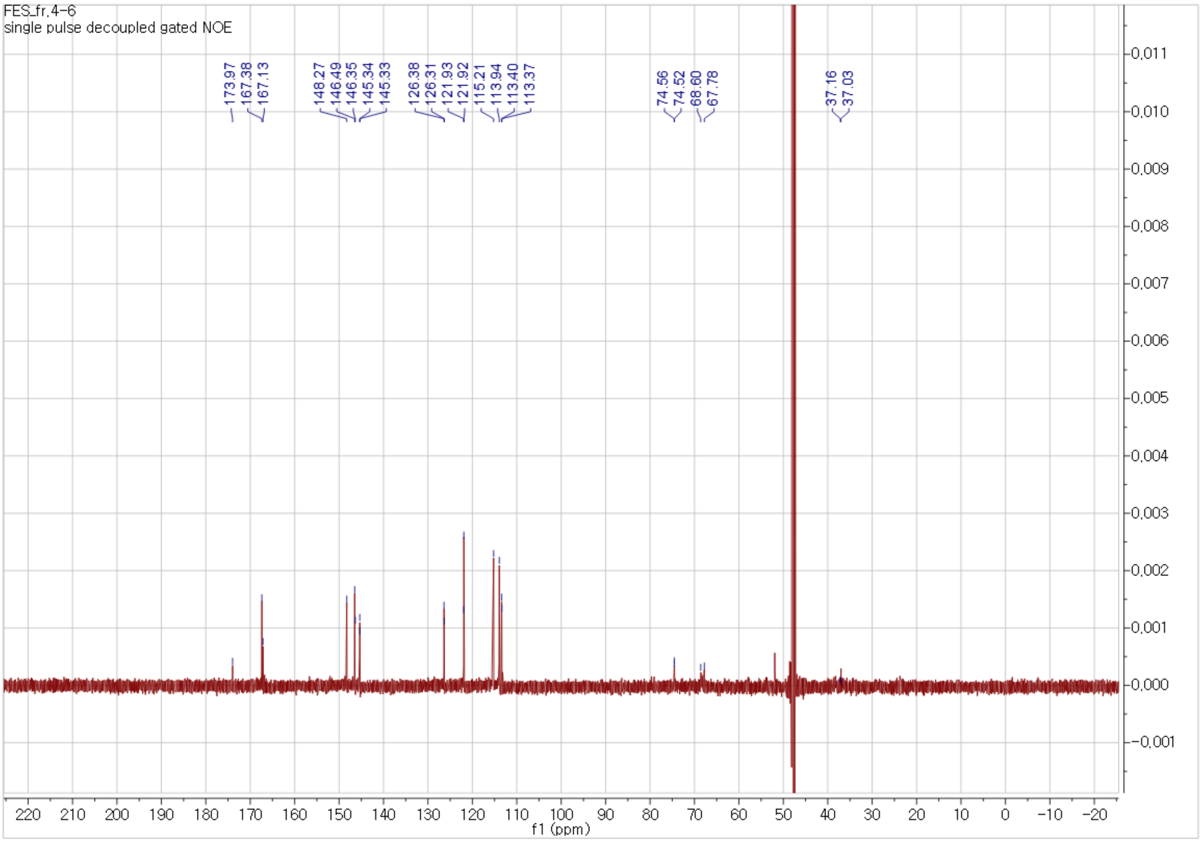

Figure S9-2: ^{13}C-NMR spectrum of compound **9** (150 MHz, CD_3OD+D_2O)

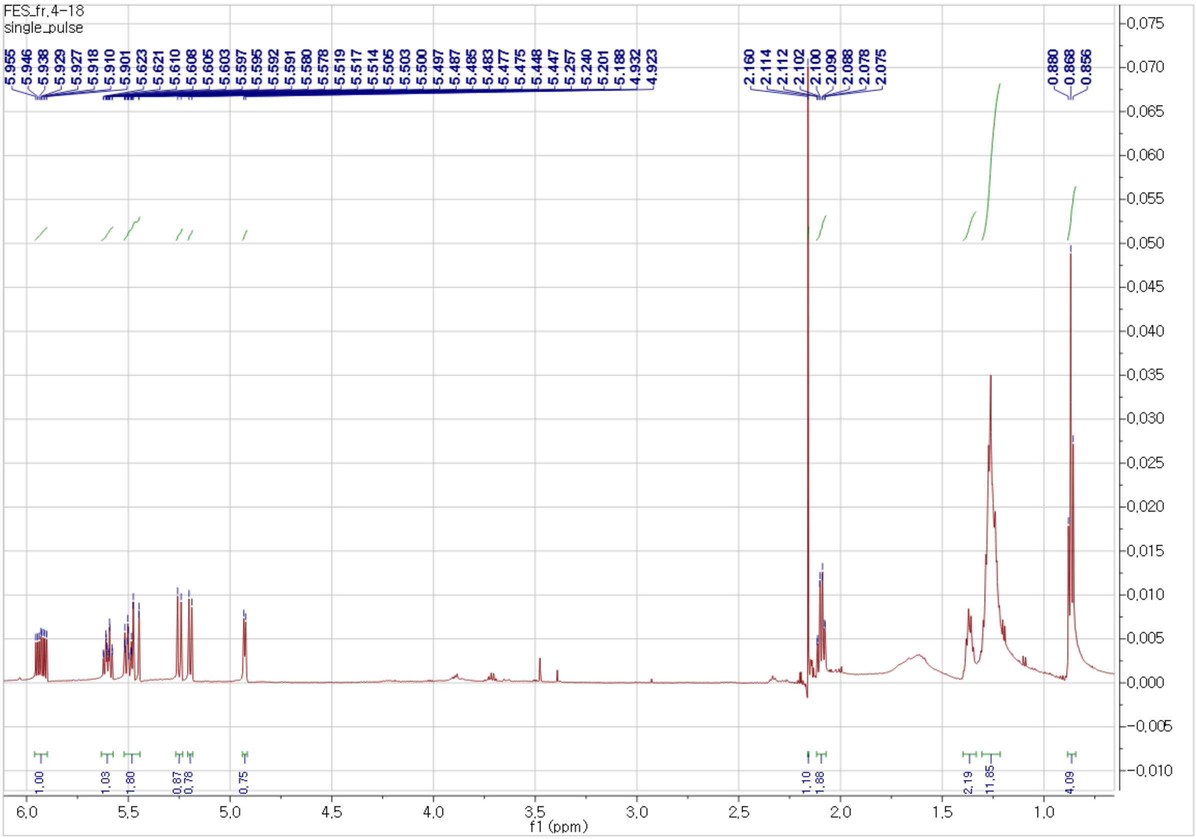

Figure S10-1: ^{1}H-NMR spectrum of compound **10** (600 MHz, $CDCl_3$)

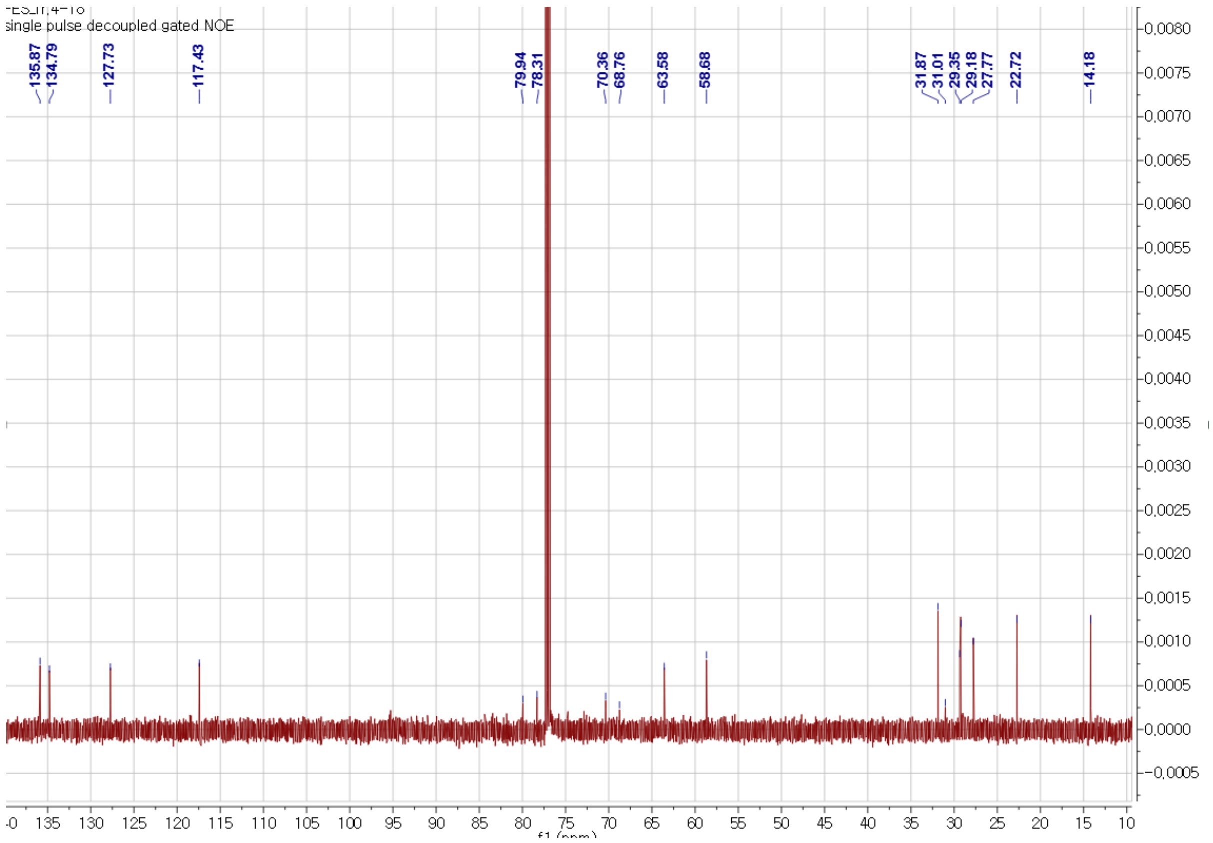

Figure S10-2: ^{13}C-NMR spectrum of compound **10** (150 MHz, $CDCl_3$)
